# Ethnobotanical study on medicinal plants used by Mulam people in Guangxi, China

**DOI:** 10.1186/s13002-020-00387-z

**Published:** 2020-07-02

**Authors:** Renchuan Hu, Chunrui Lin, Weibin Xu, Yan Liu, Chunlin Long

**Affiliations:** 1 Guangxi Institute of Traditional Medical and Pharmaceutical Sciences, Nanning, 530022 China; 2 Guangxi Key Laboratory of Traditional Chinese Medicine Quality Standards (Guangxi Institute of Traditional Medical and Pharmaceutical Sciences), Nanning, 530022 China; 3 Guangxi Institute of Botany, Guangxi Region and Chinese Academy of Sciences, Guilin, 541006 China; 4 College of Life and Environmental Sciences, Minzu University of China, Beijing, 100081 China; 5 Key Laboratory of Ethnomedicine (Minzu University of China), Ministry of Education, Beijing, 100081 China; 6 Kunming Institute of Botany, Chinese Academy of Sciences, Kunming, 650201 China

**Keywords:** Medicinal plants, Mulam people, Traditional medicinal knowledge, Luocheng County

## Abstract

**Background:**

The Mulam are an ethnic group native to Guangxi, and nearly 80% of the Mulam population lives in Luocheng Mulam Autonomous County, northern Guangxi, southern China. They have accumulated rich medicinal folk knowledge through practice and experience in their long-term struggles with disease and the harsh natural environment. However, their traditional medicinal knowledge is threatened due to a lack of written records, conservative inheritance patterns, and rapid economic development. Therefore, the investigation and documentation of medicinal plants and their associated indigenous wisdom are necessary.

**Method:**

Ethnobotanical data were collected from 12 villages and five communities in Luocheng County from January 2013 to April 2017. A total of 128 informants were interviewed through semistructured interviews, field observations, group discussions, and guided field walks. Quantitative indices such as use categories, preference ranking exercises, the informant consensus factor (ICF), and the fidelity level (FL) were used to evaluate the importance of medicinal plant species. Additionally, group discussions were conducted about the conservation of and threats to medicinal plants and traditional knowledge.

**Results:**

A total of 456 medicinal plant species from 350 genera and 132 families were recorded and documented in our ethnobotanical investigation. Most of them (335 species, 73.47%) were obtained from wild habitats. Most of the documented species (246) were herbaceous (54%), followed by shrubs, with 76 species (17%), lianas, with 75 species (16%), and trees, with 59 species (13%). The most common method of administration was oral administration, which was used for 390 species (62.70%). The most common method of preparation was decoction (316 species, 54.11%). The plants were used to treat 312 human diseases in 12 disease categories, and most of the categories had a high ICF value. The highest ICF value was recorded for gynecological ailments (0.92), followed by nervous and psychosomatic problems (0.90) and digestive system diseases (0.89). Traditional medicinal knowledge and medicinal plants are under threat due to conservative inheritance processes and anthropogenic pressures for various reasons.

**Conclusion:**

A rich diversity of medicinal plants is distributed in the Mulam area, and these plants play an important role in healthcare among the Mulam people. Mulam people are skilled in using the plants in their surroundings to treat diseases in their daily lives. However, their traditional medicinal knowledge and medicinal plants are greatly threatened by rapid economic development for various reasons. Thus, policies and practices for the conservation of medicinal plants and the associated traditional knowledge are necessary.

## Background

Medicinal plants have been used for many centuries not only in rural areas but also increasingly by urban citizens in both developing and developed countries [1–7]. According to the World Health Organization (WHO), approximately 80% of populations worldwide depend on herbal medicine for their healthcare needs, especially in rural areas [8]. In developing countries, traditional medicines provide an inexpensive source of primary health care due to the lack of modern health facilities [9, 10].

Herbal medicines have been widely accepted in China since ancient times. *Shennong Bencao Jing* (Shennong’s Herbal Classic) was the first book that systematically introduced and described traditional medicinal plant knowledge in the Eastern Han Dynasty (25 AD–220 AD) [11]. Traditional medicinal plants currently play an important role in protecting people’s lives and health in ethnic minority regions, especially in remote and less-developed areas [12–17].

Guangxi is an autonomous region of ethnic minorities, with Zhuang as the main group, and of multiethnic groups living together. The herbal medicinal markets during the Dragon-Boat Festival are very famous in the Zhuang and Yao communities of Guangxi [18–20]. Most members of ethnic minorities live in mountainous or hilly areas, and they are very good at using and naming the medicinal plants in their surroundings [21–25].

The Mulam are an ethnic group native to Guangxi, with a population of more than 210,000 [26]. Nearly 80% of the Mulam people live in Luocheng Mulam Autonomous County, Guangxi [26, 27]. Mulam people believe that human beings are an organic combination of “lingqi” (the energy that sustains living organisms), blood, tissue, bone, and muscle. They advocate “the unity of nature and man,” that is, harmony among people and between people and nature, with attention paid to both physical and mental health. “The unity of nature and man” is expressed in daily life as, for example, family members of all ages poking fun each other and through collective activity, such as the lion dance, dragon dance, monkey jumping, “zoupo” (antiphonal folk song singing by young people), and so on; these activities are beneficial to mental and physical health [28]. In their long history, Mulam people have accumulated rich folk medicinal knowledge and described many unique experiences in treating common local diseases (e.g., traumatic injuries, cough, diarrhea). Mulam folk medicinal knowledge has been enriched and developed through the process of use; this knowledge plays an important role in local daily life but has not been scientifically reported or studied. In addition, traditional medicinal knowledge is greatly threatened due to the lack of a written record and to conservative inheritance patterns. Young people prefer to look for higher-income jobs in urban areas and are not interested in traditional medicinal knowledge. Therefore, the investigation and documentation of medicinal plants and the associated indigenous wisdom are necessary. This study investigated medicinal plants and related traditional knowledge of the Mulam people, analyzed their ethnic medicinal characteristics and current threats, and proposed conservation strategies.

## Methods

### Study area

**Fig. 1:**
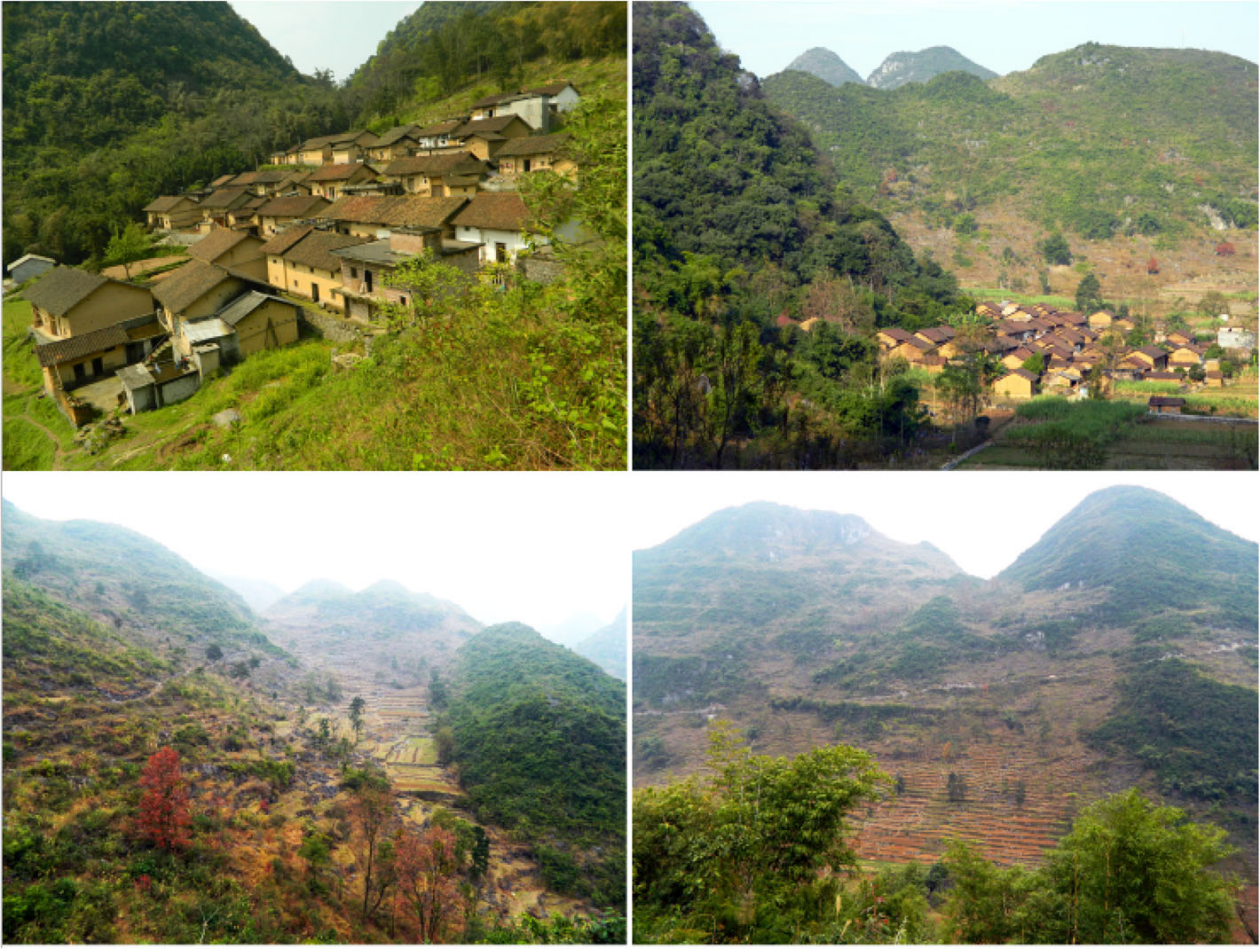
Mulam villages and the surrounding farming fields

**Fig. 2:**
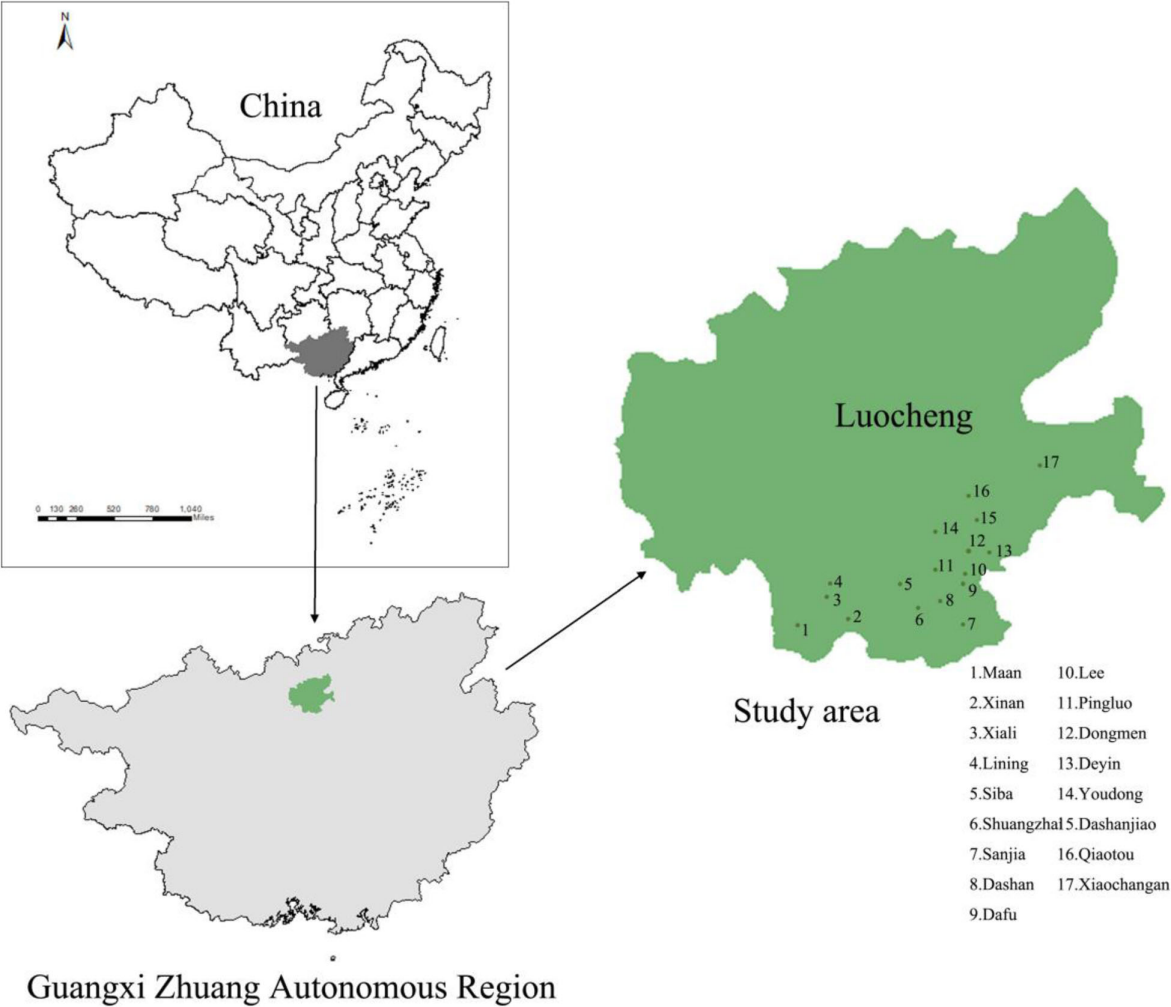
A sketch map of the study area

The study area is Luocheng Mulam Autonomous County, where the Mulam people live. Luocheng Mulam Autonomous County is situated in the subtropical zone between 24° 38′ and 25° 12′ east longitude and between 108° 29′ and 109° 10′ north latitude, with an annual average temperature of 19 °C and annual rainfall of 1566 mm. The vegetation category is the subtropical evergreen montane forest [26, 28]. Most Mulam villages are located on small strips of flat land or slopes in the karst mountainous area of southern Luocheng Mulam Autonomous County (Fig. 1). Based on the characteristics of traditional Mulam settlements and suggestions from local government officials, 12 villages (Xinan, Maan, Lining, Shuangzhai, Dashan, Youdong, Pingluo, Dafu, Lee, Dashanjiao, Deyin, Sanjia) and five townships (Dongmen, Xiali, Siba, Xiaochangan, Qiaotou) were selected as the investigation sites (Fig. 2).

Mulam settled in Luocheng during the Pre-Qin Dynasty (twenty-first century BC–221 BC) [26, 27]. The Mulam language is part of the Dong-Shui branch of the Zhuang-Dong language group in the Chinese-Tibetan language family. The Mulam language has its own independent and complete language system and preserves the language of the ancient Yue people [29]. Mulam people have multiple beliefs. They believe that every village or region is protected by a deity, so they have constructed temples around their villages, such as “Shewang,” “Powang,” “Tuzhu,” “Zaowang,” and “God of Mountain” [28]. They also believe in Taoism and Buddhism. They grow rice, corn, and potatoes as staple foods. Cats and snakes are their taboo foods. Most Mulam people engage in traditional agriculture and can identify common herbal medicines and treat common diseases. For example, they use *Artemisia argyi* for traumatic injuries, *Lobelia chinensis* for wound healing, *Sarcandra glabra* for the common cold, and so on [28].

### Ethnobotanical data collection

A total of 128 informants (81 males and 47 females) were interviewed in the study area. Among them, 84 informants were selected using the snowball method from the herbal medicinal market and Mulam villages, and 44 key informants were selected purposively and systematically after visiting local officers, village leaders, agricultural technicians, and other people in the study area via a reconnaissance survey prior to data collection. Local healers were automatically qualified as key informants who are custodians of indigenous knowledge of medicinal plants [30]. The informants were local inhabitants aged between 32 and 86 years old. Before each interview, prior informed consent was requested, and throughout the study, international codes of ethics were respected. After obtaining consent, various strata of participants (traditional healers, farmers, village leaders, religious leaders, and health officials) were interviewed.

Ethnobotanical data were collected from January 2013 to April 2017. Information about the medicinal use of plants was collected through semistructured interviews, observations, field visits, and group discussions in the investigation area [22, 31–33]. Interviews and discussions were performed based on a checklist of questions prepared in Chinese and translated into the Mulam language. The local names of the plants, the ailments treated by the plants, the plant parts used, the condition of the plant material, the modes of preparation, and the routes of administration were carefully recorded during the interviews with the informants. Vegetation categorization information was also requested and recorded. Other information, including the name, age, occupation, and education level of the informants, was collected in detail. Furthermore, we also recorded the geographic locality and date of the interview. Group discussions were conducted about the conservation of and threats to medicinal plants and traditional knowledge. In addition, the key informants were asked to perform preference ranking exercises.

### Specimen collection and identification

Field observations were performed with traditional healers to identify the morphological features and habitats of each medicinal plant species. Voucher specimens and photographs of the local medicinal plants were collected from the field and from home gardens, and the habits and habitats of these plants were recorded. For future reference, voucher specimens were made and deposited in the Herbarium of Guangxi Institute of Botany (IBK), Guangxi Zhuang Autonomous Region and Chinese Academy of Sciences, Guilin, Guangxi, China.

Voucher specimens and photographs were identified and confirmed according to *Flora of China*, *Flora of Guangxi*, and botanical websites (e.g., http://www.tropicos.org/, http://www.cvh.ac.cn/search, http://www.plant.csdb.cn/). Finally, the identified specimens were reaffirmed by taxonomic experts from IBK, and the inventory of medicinal plants was completed.

### Data analysis

Data analysis was carried out by using ethnobotanical investigation and descriptive statistical methods, such as frequency and percentage, to evaluate the importance of the plant species mentioned in the study area.

Preference ranking exercises [32–34] were conducted by asking informants to rank the most important medicinal plants that were frequently used by the local people based on their preference and the importance of the plant in the community. The plants in this exercise were shortlisted by the key informants, and then their importance in managing diseases was discussed. The ranking was based on the efficacy of the medicinal plants. If a medicinal plant was believed to be the most effective for a disease, it was given the highest value of 10 for the selected disease. In contrast, the least-effective plant would be given a value of 1. Each plant species was given a ranking based on its total score. The total ranking for the preference exercise was obtained by summing the number of informants who participated [28].

The informant consensus factor (ICF) was calculated to determine the effectiveness of the medicinal plants in each ailment category according to Heinrich et al. [31]. The formula is provided below:

$$ \mathrm{IFC}=\left(\mathrm{nur}-\mathrm{nt}\right)/\left(\mathrm{nur}-1\right) $$

nur is the number of individual reports of a plant use for a particular illness category and nt is the total number of species used by all informants for this illness category.

The fidelity level (FL) was calculated for each of the 15 preferred species for their popularity according to the key informants who cited them in the treatment of particular ailments [31, 35, 36]. The formula is provided below:

$$ \mathrm{FL}=\frac{{\mathrm{I}}_{\mathrm{p}}}{{\mathrm{I}}_{\mathrm{u}}}\times 100\% $$

*I*_p_ is the number of informants who suggested the use of a species for the same major purpose (therapeutic use) and *I*_u_ is the total number of informants who mentioned the plant species for any use.

## Results

### Demographics of the informants

A total of 128 informants, 84 of whom were general informants and 44 of whom were key informants, from Luocheng County agreed to participate in this study. The distribution of informants by age, gender, and education level is shown in Table 1. The age of the informants ranged from 32 to 86 years old. Among them, 82.3% of informants were over 40 years old, 58.59% of informants had only a primary education, and 12.5% were illiterate. There were more male informants (81, 63.28%) than female informants.

**Table 1:** Demographic profile of informants

Indicator	Description	General informants	Key informants	Total	Frequency (%)
Age	30–39	12	1	13	10.16
	40–49	23	4	27	21.09
	50–59	25	17	42	32.81
	60–69	12	16	28	21.88
	70–79	7	3	10	7.81
	≧ 80	5	3	8	6.25
Gender	Male	43	38	81	63.28
	Female	41	6	47	36.72
Education	Illiteracy	11	5	16	12.50
	Primary	53	22	75	58.59
	Secondary	20	13	33	25.78
	Tertiary	0	4	4	3.13

### Medicinal plants recorded

From the study sites, a total of 456 medicinal plant species belonging to 350 genera and 132 families were documented. Ethnomedicinal information for each species, including its scientific name, Chinese name, Mulam name, family name, habit, habitat, plant parts used, cited sources, preparation, and use, is listed in Table 2.

**Table 2:** Inventory of medicinal plants traditionally used by Mulam people

Scientific name	Chinese name	Mulam name	Family	Habit	Habitat	Parts used	Preparation and uses	Cited sources
*Abelmoschus sagittifolius* (Kurz) Merr.	Jianyeqiukui箭叶秋葵	–	Malvaceae	Herb	Home garden	Whole plant	Decoction; taken orally for kidney deficiency, backache	451225130608007
*Abrus cantoniensis* Wall. ex Wightet Arn.	Guangdongxiangsizi广州相思子	hɣɔk^8^ci^1^kwət^7^	Fabaceae	Herb	Wild	Whole plant	Decoction; taken orally for jaundice hepatitis, stomachache, scrofula. Pounded fresh part applied on the affected area, treating for traumatic injury, painful swelling	451225130719008
*Acalypha australis* L.	Tiexiancai铁苋菜	–	Euphorbiaceae	Herb	Wild	Whole plant	Decoction; taken orally for clearing away heat and promoting diuresis. Pounded fresh part applied on the affected area, treating for hemostasis with astringents	451225130517008
*Achyranthes aspera* L.	Tuniuxi土牛膝	–	Amaranthaceae	Herb	Wild	Whole plant	Decoction; taken orally or medicinal bath for clearing away heat and toxic material, diuresis, treating for ascites, nephritis, sweating	451225130517018
*Achyranthes bidentata* Blume	Niuxi牛膝	mai^4^cen^1^tən^2^	Amaranthaceae	Herb	Wild	Root, Rhizome	Medicinal liquor; taken orally for treating rheumatism, traumatic injury. Decoction; taken orally for sore throat, urinary urgency, dysuria, furuncle and carbuncle	451225130101019
*Achyranthes longifolia* Makino	Liuyeniuxi柳叶牛膝	–	Amaranthaceae	Herb	Wild	Whole plant	Decoction; taken orally for calculosis	451225130517052
*Aconitum carmichaelii* Debeaux	Wutou乌头	–	Ranunculaceae	Herb	Home garden	Whole plant	Pounded fresh part applied on the affected area, treating for hyperosteogeny	451225130607001
*Acorus calamus* L.	Changpu菖蒲	–	Acoraceae	Herb	Wild	Whole plant	Decoction; medicinal bath, treating for carbuncle, headache	451225130607020
*Acorus gramineus* Soland.	Jinqianpu金钱蒲	sik^8^tshja:ŋ^1^pu^2^	Acoraceae	Herb	Wild	Rhizome	Decoction; taken orally for epilepsy, phlegm heat, abdominal distension, abdominal pain. Powdered, applied on the affected area for traumatic injury	451225130310058
*Adina rubella* Hance	Xiyeshuituanhua细叶水团花	–	Rubiaceae	Shrub	Wild	Root	Decoction; taken orally for treating tracheitis	451225130310001
*Agrimonia pilosa* Ledeb.	Longyacao龙芽草	ma^4^ljen^6^a:n^1^	Rosaceae	Herb	Wild	Whole plant	Decoction; taken orally for stanching bleeding, cool the blood, dissipate blood stasis, diarrhea	451225130719003
*Akebia trifoliata* (Thunb.) Koidz. subsp. *australis* (Diels) T. Shimizu	Baimutong白木通	–	Lardizabalaceae	Liana	Wild	Stem	Decoction; taken orally for nephritis	451225130428026
*Alangium chinense* (Lour) Harms.	Bajiaofeng八角枫	pa:t^7^kak^7^foŋ^1^	Cornaceae	Shrub	Wild	Stem, Leaf, Root	Decoction; medicinal bath for treating rheumatism, numbness of limbs, internal lesion caused by overexertion, traumatic injury, dissipate blood stasis, relieve pain	451225130421036
*Albizia julibrissin* Durazz.	Hehuan合欢	thəu^5^mu^2^kwa:n^1^tɔ^1^	Fabaceae	Tree	Wild	Bark	Decoction; taken orally for treating restlessness, insomnia and dreaminess, ADHD. Pounded fresh part applied on the affected area, treating for abscess, traumatic injury	451225130430033
*Alchornea trewioides* (Benth.) Muell. Arg.	Hongbeishanmagan红背山麻杆	–	Euphorbiaceae	Shrub	Wild	Stem and leaf	Medicinal bath, treating for eczema	451225130307019
*Alisma orientale* (Samuel) Juz.	Dongfangzexie东方泽泻	–	Alismataceae	Herb	Wild	Whole plant	Decoction; taken orally for enteritis	451225131107033
*Allium fistulosum* L.	Cong葱	thɔŋ^1^pa:k^8^	Amaryllidaceae	Herb	Home garden	Bulb	Decoction; taken orally for typhoid, headache, abdominal pain, constipation, urinary stoppage, diarrhea, abscess	451225131107034
*Allium macrostemon* Bge.	Yongbai薤白	kɣo^3^ceu^4^	Amaryllidaceae	Herb	Wild	Stem	Decoction; taken orally for thoracic obstruction, diarrhea. Pounded fresh part applied on the affected area, treating for furuncle	451225130729012
*Allium sativum* L.	Suan蒜	kɣo^3^hɣɔ^2^	Amaryllidaceae	Herb	Home garden	Bulb	Decoction; taken orally or medicinal bath for treating fever, headache, angina, hiccough, anorexia, poor appetite, furuncle, carbuncle	451225130311031
*Allium tuberosum* Rottler ex Spreng.	Jiu韭	ha:i^5^la:k^8^	Amaryllidaceae	Herb	Home garden	Seed, Leaf	Fried; taken orally directly for treating impotence, nocturnal emission, frequent micturition, enuresis, diarrhea, leukorrhea, turbidity, infantile convulsion	451225130723008
*Alocasia cucullata* (Lour.) Schott	Jianweiyu尖尾芋	–	Araceae	Herb	Wild	Rhizome	Decoction after slicing and drying; taken orally for hepatocirrhosis	451225130425003
*Aloe vera* (L.) Burm. f.	Luhui芦荟	ma^1^ləm^6^	Xanthorrhoeaceae	Herb	Home garden	Leaf juice	Taken orally directly for treating constipation, infantile convulsion, infatile malnutrition with fever, ringworm, hemorrhoid complicated by anal fistula, scrofula	451225131107017
*Alyxia sinensis* Champ. ex Benth.	Lianzhuteng链珠藤	–	Apocynaceae	Liana	Wild	Stem, Leaf	Decoction; taken orally for treating bladder cancer, uterine cancer	451225130807002
*Amaranthus spinosus* L.	Cixian刺苋	–	Amaranthaceae	Herb	Home garden	Root	Pounded fresh part applied on the affected area, treating for piles	451225130606018
*Amomum tsaoko* Crevost et Lemarie	Caoguo草果	–	Zingiberaceae	Herb	both	Seed	Taken orally directly for aid digestion	451225130728017
*Amorphophallus konjac* K. Koch	Huamoyu花磨芋	ɣa:k^7^la:i^4^	Araceae	Herb	both	Tuber	Decoction after slicing and drying; taken orally for cough. Powdered, applied on the affected area, treating for traumatic injury, furuncle, erysipelas	451225130519009
*Amygdalus persica* L.	Tao桃	hwi^1^tɔ^2^la:k^8^	Rosaceae	Tree	Home garden	Seed	Taken orally directly for treating dysmenorrhea, abdominal pain, traumatic injury, abscess of lung, intestinal carbuncle, constipation due to intestinal dryness	451225130424017
*Andrographis paniculata* (Burm. f.) Nees	Chuanxinlian穿心莲	tshjøn^5^təm^1^ljen^2^	Acanthaceae	Herb	Wild	Whole plant	Decoction; taken orally for influenza, sore throat, tracheitis, pneumonia	451225121230008
*Anredera cordifolia* (Ten.) Steenis	Luokuishu落葵薯	–	Basellaceae	Herb	Home garden	Tuber, Stem and leaf	Stewed with pork bone and drunk the soup for supplementary blood and nutrition. Pounded fresh part applied on the affected area for dissipate blood stasis	451225121230001
*Arachis hypogaea* L.	Luohuasheng落花生	ti^6^tau^6^	Fabaceae	Herb	Home garden	Seed	Taken orally directly for treating irritating dry cough, stomachache, hypertension, dizziness due to deficiency of blood	451225130606021
*Aralia chinensis* L.	Huangmaocongmu黄毛楤木	khai^1^mai^4^	Araliaceae	Shrub	Wild	Bark or Stem	Decoction; taken orally or medicinal bath, treating for rheumatic arthritis, nephritis edema, ascites due to cirrhosis, hepatitis, stomachache, turbidity, metrorrhagia, traumatic injury, abscess	451225130518021
*Arctium lappa* L.	Niupang牛蒡	tən^2^ha^5^la:k^8^	Asteraceae	Herb	both	Fruit	Decoction; taken orally for treating wind-heat type common cold, cough, sore throat, eczema	451225130428019
*Ardisia crenata* Sims	Zhushagen朱砂根	–	Primulaceae	Shrub	Wild	Root, Stem	Medicinal liquor; taken orally or applied on the affected area, treating for rheumatism	451225130308012
*Ardisia gigantifolia* Stapf.	Zoumatai走马胎	ça:u^1^tsha:m^3^ma^4^	Primulaceae	Shrub	Wild	Root	Medicinal liquor; taken orally or applied on the affected area, treating for rheumatism, dispelling wind, remove dampness, removing blood stasis, traumatic injury, waist-leg weakness, carbuncle ulcer	451225130610040
*Ardisia lindleyana* D. Dietr.	Xiaoluosan小罗伞	mai^4^ta:n^5^niŋ^5^	Primulaceae	Shrub	Wild	Root or Whole plant	Decoction; taken orally or medicinal bath for treating rheumatoid arthritis, amenorrhea, dysmenorrhea. Pounded fresh part applied on the affected area, treating for traumatic injury	451225130311035
*Ardisia japonica* (Thunb) Blume	Zijinniu紫金牛	te^3^ti^6^tsa^2^	Primulaceae	Shrub	Wild	Stem, Leaf	Decoction; taken orally and medicinal bath for treating chronic bronchitis, tuberculosis, nephritis, hypertension, swollen toxin, hernia	451225130722002
*Areca catechu* L.	Binglang槟榔	–	Arecaceae	Tree	Home garden	Rhizome	Decoction; taken orally for liver ascites	451225130610033
*Arisaema erubescens* (Wall.) Schott	Yibasannanxing一把伞南星	–	Araceae	Herb	Wild	Root	Decoction; taken orally for clearing away heat and toxic materials	451225130311032
*Aristolochia debilis* Sieb. et Zuce.	Madongling马兜铃	mai^4^həu^1^mɣa:ŋ^1^	Aristolochiaceae	Liana	Wild	Root	Decoction; taken orally for relieve pain, detoxifcation detumescence, blood pressure lowering	451225130729011
*Aristolochia fordiana* Hemsl.	Tongchenghu通城虎	–	Aristolochiaceae	Liana	both	Whole plant	Taken orally directly for anti-inflammatory, gastritis, enteritis. Pounded fresh part applied on the affected area, treating for snake bite	451225121204039
*Armeniaca mume* Sieb.	Mei梅	u^5^məi^6^	Rosaceae	Tree	Home garden	Fruit	Taken orally directly for treating diarrhea, hemafecia, cough with lung heat, sore throat, depriving ascarid	451225130426040
*Artemisia anomala* S. Moore.	Qihao奇蒿	pɛ:k^8^hwa^1^wəi^1^	Asteraceae	Herb	Wild	Whole plant	Taken orally directly for treating amenorrhea, abdominal distention, postpartum blood stasis. Pounded fresh part applied on the affected area, treating for traumatic injury, carbuncle toxin	451225130427037
*Artemisia argyi* H. Lév. et Vaniot	Ai艾	ŋa:i^6^fa^5^	Asteraceae	Herb	Wild	Leaf	Moxibustion; Treating for tocolysis, dysmenorrhea, irregular menses, leukorrhea, metrorrhagia and metrostaxis	451225130720008
*Artemisia capillaris* Thunb.	Yinchenhao茵陈蒿	mau^5^hɣɔk^8^	Asteraceae	Herb	Wild	Stem and leaf	Taken orally directly for treating damp and hot jaundice, dysuria, sores	451225130102009
*Artemisia carvifolia* Buch.-Ham. ex Roxb.	Qinghao青蒿	ŋa:i^6^həu^1^	Asteraceae	Herb	Wild	Whole plant	Decoction; taken orally or medicinal bath for treating malaria, diarrhea, jaundice. Pounded fresh part applied on the affected area, treating for scabies, pruritus	451225130610003
*Artemisia indica* Willd.	Wuyueai五月艾	–	Asteraceae	Herb	Wild	Whole plant	Decoction; medicinal bath for dispelling wind and removing dampness	451225130427028
*Artemisia scoparia* Waldst. et Kit.	Zhumaohao猪毛蒿	–	Asteraceae	Herb	Wild	Whole plant	Taken orally directly for treating stomachache	451225130518018
*Arundo donax* L.	Luzhu芦竹	–	Poaceae	Shrub	Wild	Root	Decoction; taken orally for pharyngitis, nephritis, edema	451225130611004
*Asarum caudigerum* Hance	Weihuaxixin尾花细辛	–	Aristolochiaceae	Herb	Wild	Whole plant	Pounded fresh part applied on the affected area, for relieve pain, toothache, gout	451225130309040
*Asparagus cochinchinensis* (Lour.) Merr	Tianmendong天门冬	mən^6^tɔŋ^1^	Asparagaceae	Herb	Wild	Rhizome	Decoction; taken orally for cough, hemoptysis, pneumalgia, sore throat	451225130428020
*Bauhinia championii* (Benth.) Benth.	Longxuteng龙须藤	ça:u^1^ma^6^jin^5^	Fabaceae	Liana	Wild	Stem	Medicinal liquor; taken orally or applied on the affected area, treating for gastritis, rheumatism, traumatic injury, bone fracture	451225121231022
*Belamcanda chinensis* (L.) DC.	Shegan射干	məm^6^kwət^7^hɣɔk^8^	Iridaceae	Herb	Wild	Rhizome	Decoction; taken orally for sore throat, abscess, amenorrhea	451225130428054
*Benincasa hispida* (Thunb.) Cogn.	Donggua冬瓜	tɔŋ^5^kwa^1^ŋɣa^2^	Cucurbitaceae	Liana	Home garden	Peel	Decoction; taken orally for nephritis edema, poor urination	451225130430039
*Bidens bipinnata* L.	Popozhen婆婆针	la:i^4^tshəm^1^hɣɔk^8^	Asteraceae	Herb	Wild	Whole plant	Decoction; taken orally for acute appendicitis, mastalgia, bacillary dysentery, angina, kidney deficiency, backache, nephritis, migraine. Pounded fresh part applied on the affected area, treating for snake bite, traumatic injury	451225130608021
*Bidens pilosa* L.	Guizhencao鬼针草	–	Asteraceae	Herb	Wild	Stem and leaf	Decoction; medicinal bath for degerming and anti-inflammatory	451225130608026
*Bischofia javanica* Blume	Qiufeng秋枫	–	Euphorbiaceae	Tree	both	Root	Pounded fresh part applied on the affected area, treating for piles	451225131108037
*Bletilla formosana* (Hayata) Schltr.	Xiaobaiji小白及	–	Orchidaceae	Herb	Wild	Tuber	Stewed with pork bag and taken orally directly for tumour	451225130309006
*Bletilla striata* (Thunb. ex A. Murray) Rchb. f.	Baiji白及	–	Orchidaceae	Herb	Wild	Tuber	Decoction; taken orally for gastric ulcer, tuberculosis	451225130307037
*Boehmeria nivea* (L.) Gaudich.	Zhuma苎麻	pə^6^ma^6^ta:ŋ^1^	Urticaceae	Shrub	Wild	Root	Decoction; taken orally for internal hemorrhage, hemokelidosis, threatened abortion, poor urination. Pounded fresh part applied on the affected area, treating for poisoned sore, snake and insect injury	451225130421030
*Botrychium lanuginosum* Wall.	Rongmaoyindijue绒毛阴地蕨	–	Ophioglossaceae	Herb	Wild	Whole plant	Decoction; taken orally for lunacy, settle fright and quiet the spirit	451225131107031
*Brassica juncea* (L.) Czern.	Jiecai芥菜	–	Brassicaceae	Herb	Home garden	Whole plant	Decoction; taken orally for calculosis	451225130307031
*Bryophyllum pinnatum* (L. f. ) Oken	Luodishenggen落地生根	–	Crassulaceae	Herb	Wild	Whole plant	Pounded fresh part applied on the affected area, treating for detumescence relieve pain, detoxicating and generating muscles	451225130607009
*Buchnera cruciata* Buch. Mutis ex. L. f. Hamilt.	Heicao黑草	hɣɔk^8^nam^1^	Orobanchaceae	Herb	Wild	Whole plant	Decoction; taken orally or medicinal bath for treating eruptive disease, typhoid, epilepsy, painful swelling	451225130310048
*Buddleja officinalis* Maxim.	Mimenghua密蒙花	–	Scrophulariaceae	Shrub	Wild	Root	Decoction; taken orally for ascites due to cirrhosis, jaundice hepatitis	451225130310013
*Callerya reticulata* (Benth.) Schot	Wangluojixueteng网络鸡血藤	–	Fabaceae	Liana	Wild	Stem	Medicinal liquor; taken orally or rinsed, treating for rheumatism, free the channels and network vessels, osteoporosis	451225130722005
*Callerya speciosa* (Champ. ex Benth.) Schot	Meilijixueteng美丽鸡血藤	–	Fabaceae	Liana	Wild	Whole plant	Medicinal liquor; taken orally for treating tracheitis, osteoporosis	451225130607039
*Callicarpa macrophylla* Vahl	Dayezizhu大叶紫珠	–	Verbenaceae	Shrub	Wild	Stem and leaf	Pounded fresh part applied on the affected area, treating for protrusion of lumbar intervertebral disc, hyperosteogeny, rheumatism	451225130607013, 451225130722004
*Camellia oleifera* Abel	Youcha油茶	tsa:i^6^jəu^2^	Theaceae	Tree	Wild	Oil from seeds	Taken orally directly treating for abdominal pain, depriving ascarid, intestinal dryness and nodding. Applied on the affected area, treating for scabies, scald	451225130421041
*Campanumoea javanica* Blume Bijdr.	Jinqianbao金钱豹	–	Campanulaceae	Herb	Wild	Root	Decoction; taken orally for lung heat, dry cough	451225130608018
*Canarium album* (Lour.) Rauesch.	Ganlan橄榄	ka:n^3^la:n^3^	Burseraceae	Tree	Home garden	Fruit	Taken orally directly for sore throat, cough hemoptysis, bacillary dysentery, alleviate a hangover	451225130609002
*Canna indica* L.	Meirenjiao美人蕉	tɔŋ^6^fa^5^	Cannaceae	Herb	Wild	Stem, Flower	Decoction; taken orally for acute jaundice hepatitis, protracted dysentery, leukorrhea, irregular menses, hypertension. Pounded fresh part applied on the affected area, abscess	451225130518003
*Canscora lucidissima* (Levl. et Vant.) Hand.-Mazz.	Chuanxincao穿心草	hɣɔk^8^tshjøn^5^təm^1^	Gentianaceae	Herb	Wild	Whole plant	Decoction; taken orally for hepatopathy, cough with lung heat, hepatitis, jaundice, pectoralgia, stomachache, traumatic injury	451225130311007
*Capsella bursa-pastoris* (L.) Medic.	Jicai荠菜	ma^1^ja^4^	Brassicaceae	Herb	Home garden	Whole plant	Decoction; taken orally for diarrhea, edema, gonorrhea, internal hemorrhage, red eyes painful swelling	451225130608022
*Cardiospermum halicacabum* L.	Daodiling倒地铃	–	Sapindaceae	Herb	Wild	Whole plant	Taken orally directly or pounded fresh part applied on the affected area for expelling parasite, relieve pain	451225130519053
*Carica papaya* L.	Fanmugua番木瓜	–	Caricaceae	Tree	Home garden	Peel	Stewed with pork bone and drunk the soup, treating for osteoporosis	451225130312001
*Cassytha filiformis* L.	Wugenteng无根藤	ça:u^1^khu^5^mɛ^2^ni^4^	Lauraceae	Herb	Wild	Whole plant	Decoction; taken orally for diuresis, detumescence, cough with lung heat, jaundice, diarrhea, internal hemorrhage, abscess, scabies, scald	451225130311062
*Catalpa ovata* G. Don	Zi梓	–	Bignoniaceae	Tree	Wild	Fruit	Decoction; taken orally for hepatopathy	451225130424024
*Cayratia albifolia* C. L. Li	Baimaowulianmei白毛乌蔹莓	ça:u^1^mu^5^mai^4^	Vitaceae	Liana	Wild	Root, Leaf	Root: medicinal liquor; taken orally for treating rheumatic arthritis. Leaf: pounded fresh part applied on the affected area, treating for unknown swollen toxin; Chewing, treating for toothache.	451225130426036
*Cayratia japonica* (Thunb.) Gagnep.	Wulianmei乌蔹莓	ŋɔ^4^fa^5^mwa:i^2^	Vitaceae	Liana	Wild	Whole plant	Decoction; taken orally for rheumatoid arthritis, jaundice, diarrhea, hematuria, gonorrhea, furuncle abscess, erysipelas	451225130606003
*Celastrus orbiculatus* Thunb.	Nansheteng南蛇藤	ta^6^pɣa^1^lɔŋ^2^	Celastraceae	Liana	Wild	Stem	Decoction; taken orally for arthralgia and myalgia, numbness of limbs, infantile convulsion, measles syndrome, diarrhea	451225130430008
*Celosia argentea* L.	Qingxiang青葙	ja^4^ci^1^kon^1^hwa^1^	Amaranthaceae	Herb	both	Seed	Medicinal bath for insecticidal	451225130518039, 451225130608024
*Celosia cristata* L.	Jiguanhua鸡冠花	ci^1^kon^1^hwa^1^	Amaranthaceae	Herb	Home garden	Inflorescence	Decoction; taken orally for internal hemorrhage, leukorrhea	451225130607049
*Centella asiatica* (L.) Urb.	Jixuecao积雪草	chøt^7^pa:k^7^won^3^	Apiaceae	Herb	Wild	Whole plant	Decoction; taken orally for prostatitis, eruptive disease, diarrhea, jaundice, internal hemorrhage, measles. Pounded fresh part applied on the affected area, treating for furuncle abscess, traumatic injury	451225130424011
*Centipeda minima* (L.) A. Br. et Aschers.	Shihusui石胡荽	hɣɔk^8^ŋa:n^6^khu^5^tsa:n^1^	Asteraceae	Herb	Wild	Whole plant	Decoction; taken orally for dissipate blood stasis, dispelling wind detumescence, hepatitis, common cold, pharyngitis, pertussis cough, diarrhea, malaria, nasosinusitis, hemorrhoids	451225130611010
*Cephalotaxus fortunei* Hook.	Sanjianshan三尖杉	tau^6^la:n^3^sa^1^	Cephalotaxaceae	Tree	Wild	Stem and leaf	Decoction; taken orally for dry cough, dry pharynx	451225130430030
*Chenopodium hybridum* L.	Zapeili杂配藜	phɣə:t^7^nən^1^jəu^1^	Amaranthaceae	Herb	Wild	Whole plant	Decoction; taken orally for sore abscess, irregular menses, internal hemorrhage, enteritis, bacillary dysentery	451225130425013
*Chloranthus henryi* Hemsl.	Kuanyejinsulan宽叶金粟兰	ti^5^phjen^5^ŋwa^4^	Chloranthaceae	Herb	Wild	Whole plant	Pounded fresh part applied on the affected area, treating for rheumatism, arthralgia and myalgia, traumatic injury	451225130723006
*Choerospondias axillaris* (Roxb.) B. L. Burtt et A. W. Hill	Nansuanzao南酸枣	–	Anacardiaceae	Tree	Wild	Root	Decoction; taken orally for encephalemia	451225130426037
*Chrysanthemum indicum* L.	Yeju野菊	cy^6^hwa^1^ja^4^	Asteraceae	Herb	Wild	Flower	Decoction; taken orally for anti-inflammatoryy, enteritis, rheumatism, wind-heat type common cold, pneumonia, diphtheritis, hypertension, furuncle, aptha, erysipelas, eczema	451225121205038
*Chrysopogon aciculatus* (Retz.) Trin.	Zhujiecao竹节草	–	Poaceae	Herb	Wild	Whole plant	Decoction; taken orally for diuresis detumescence, clearing away heat and toxic materials	451225130611024
*Cibotium barometz* (L.) J. Sm.	Jinmaogou金毛狗	cəm^1^mɔ^2^ŋwa^1^	Cibotiaceae	Herb	Wild	Rhizome	Decoction; taken orally for hemiplegia, backache, rheumatism, urinary frequency, spermatorrhea, leukorrhea	451225121204014, 451225130728003
*Cinnamomum camphora* (L.) Presl	Zhang樟	–	Lauraceae	Tree	both	Stem, Root	Decoction; taken orally for hepatosplenomegaly, edema, hepatitis	451225130430032
*Cipadessa baccifera* (Roth) Miq.	Huimaojiangguolian灰毛浆果楝	–	Meliaceae	Shrub	Wild	Stem and leaf	Medicinal bath for thermolysis, anti-inflammatory	451225121230031
*Cirsium chinense* Gardner et Champ.	Xiaoji小蓟	ci^1^niŋ^5^	Asteraceae	Herb	Wild	Whole plant or Root	Decoction; taken orally or medicinal bath for treating internal hemorrhage, irregular menses, damp and hot, jaundice. Pounded fresh part applied on the affected area, treating for bleeding wound, furuncle, swollen toxin	451225130422019
*Cirsium japonicum* Fisch. ex DC.	Daji大蓟	ci^1^lo^4^	Asteraceae	Herb	Wild	Whole plant or Root	Decoction; taken orally for internal hemorrhage, scald, mumps, jaundice, costalgia, intestinal carbuncle	451225130422019
*Cissus pteroclada* Hayata	Yijingbaifenteng翼茎白粉藤	ça:u^1^ti^5^teŋ^2^	Vitaceae	Liana	Wild	Stem	Medicinal liquor or decoction; taken orally for activate collaterals, rheumatoid arthritis, traumatic injury	451225130310068
*Citrullus lanatus* (Thunb.) Matsum. et Nakai	Xigua西瓜	te^1^kwa^1^ŋɣa^2^	Cucurbitaceae	Liana	Home garden	Bark	Decoction; taken orally for hotness and polydipsia, oliguresis, edema	451225130606028
*Citrus maxima* (Burm.) Merr.	You柚	–	Rutaceae	Tree	Home garden	Stem and leaf	Decoction; medicinal bath for sweating	451225130426008
*Citrus sinensis* (L.) Osbeck	Tiancheng甜橙	ka:m^5^tsən^2^ŋɣa^2^	Rutaceae	Tree	Home garden	Peel	Taken orally directly for abdominal distention, nausea, vomit	451225131108015
*Citrus tangerina* Hort. et Tanaka.	Fuju福橘	cy^6^fa^5^	Rutaceae	Tree	Home garden	Peel	Taken orally directly for costalgia, acute mastitis, lump of breast	451225140408015
*Citrus trifoliata* L.	Ji枳	tsi^2^la:k^8^	Rutaceae	Tree	Home garden	Fruit	slicing and drying, decoction; taken orally for rib expansion, dyspeptic retention, hiccup, alo laxata, rectal prolapse, uterine prolapse	451225130721012
*Clausena lansium* (Lour.) Skeels.	Huangpi黄皮	ŋɣa^2^ŋa:n^3^hwi^1^la:k^8^	Rutaceae	Tree	Home garden	Fruit	Taken orally directly for removing jaundice,hepatitis, dyspeptic retention, cough asthma	451225130422041
*Clematis chinensis* Osbeck.	Weilingxian威灵仙	hɣɔk^8^məm^4^mut^8^	Ranunculaceae	Liana	Wild	Root	Decoction; taken orally for gout, obstinate arthralgia, barbiers, malaria, tetanus, painful swelling	451225121205044
*Clerodendrum bungei* Steud.	Choumudan臭牡丹	ȵin^1^lɔ^2^ta:n^1^	Lamiaceae	Shrub	Wild	Stem, Leaf	Decoction; medicinal bath for tuberculosis, carbuncle, furuncle, eczema, piles, rectal prolapse, infantile convulsion	451225130426029
*Clerodendrum cyrtophyllum* Turcz.	Daqing大青	–	Lamiaceae	Shrub	Wild	Stem and leaf	Pounded and heated the fresh part, applied on the affected area, treating for hyperosteogeny	451225130729016
*Clerodendrum japonicum* (Thunb.) Sweet	Chengtong赪桐	–	Lamiaceae	Shrub	Wild	Stem and leaf	Decoction; medicinal bath for rheumatism	451225130606025
*Cnidium monnieri* (L.) Cusson.	Shechuang蛇床	twi^2^pho^5^la:k^8^	Apiaceae	Herb	Wild	Fruit	Medicinal liquor; taken orally for treating impotence, rheumatoid arthritis, hemorrhoids eczema. Decoction; taken orally and medicinal bath for eczema scrotum, leukorrhea, pruritus vulvae, infertility	451225130421020
*Coix lacryma-jobi* L. var. *ma-yuen* (Rom. Caill.) Stapf	Yimi薏米	hɣɔk^8^lak^8^khau^5^	Poaceae	Herb	both	Seed	Stewed; taken orally directly for dysuria, edema, inchacao, invigorating spleen, diarrhea, rheumatoid arthritis, abscess of lung, intestinal carbuncle	451225130310025
*Commelina diffusa* Burm.	Jiejiecao节节草	–	Commelinaceae	Herb	Wild	Whole plant	Decoction; taken orally for lithangiuria, clearing liver and eyesight, removing dampness	451225130519005
*Coriandrum satiuum* L.	Yuansui芫荽	jøn^6^tok^8^	Apiaceae	Herb	Home garden	Whole plant	Decoction; taken orally for measles, poor appetite, stomach cold	451225130519021
*Corydalis saxicola* Bunting	Yanhuanglian岩黄连	pa:i^2^lε^5^huŋ^6^ljen^2^	Papaveraceae	Herb	both	Whole plant	Taken orally directly for anti-inflammatory	451225130426020
*Corydalis sheareri* S. Moore	Dijinmiao地锦苗	hu^5^təm^1^mwɔ^5^	Papaveraceae	Herb	Wild	Rhizome	Taken orally directly or pounded fresh part applied on the affected area, treating for stomach heat, damp and hot jaundice, edema, traumatic injury, furuncle and carbuncle	451225130307005
*Crassocephalum crepidioides* (Benth.) S. Moore	Yetonghao野茼蒿	–	Asteraceae	Herb	Wild	Stem and leaf	Pounded fresh part applied on the affected area, treating for hyperplasia of mammary glands	451225130519023
*Crataegus pinnatifida* Bge. var. *major* N. E. Br.	Shanlihong山里红	pɣa^1^tsa^1^	Rosaceae	Tree	both	Fruit	Taken orally directly for abdominal distension, anorexia, abdominal pain	451225130729010
*Crinum asiaticum* L. var. *sinicum* (Roxb. ex Herb.) Baker	Wenshulan文殊兰	khɣɛ^1^lɔŋ^2^ma^4^	Amaryllidaceae	Herb	Wild	Leaf	Pounded fresh part applied on the affected area, treating for abscess, traumatic injury, joint pain	451225130430048
*Cucumis sativus* L.	Huanggua黄瓜	–	Cucurbitaceae	Liana	Home garden	Root, Seed	Root: Decoction; taken orally for rheumatism, removing jaundice, jaundice, hepatitis. Seed: taken orally directly for treating heart disease	451225130609003
*Cucurbita moschata* (Duch. ex Lam.) Duch. ex Poiret	Nangua南瓜	cəm^1^kwa^1^piŋ^5^	Cucurbitaceae	Liana	Home garden	Peel, pedicel, Seed	Peel: Decoction; taken orally for stone. Pedicellus cucurbitae: Decoction; taken orally for treating stone, carbuncle, furuncle, scald, threatened abortion. Seed: taken orally directly, treating for tapeworm, depriving ascarid, postpartum blood stasis, piles	451225130718020
*Cupressus funebris* Endl.	Baimu柏木	–	Cupressaceae	Tree	both	Bark	Decoction; taken orally for liver ascites	451225130517006
*Curculigo orchioides* Gaertn.	Xianmao仙茅	pɣa^1^jyn^6^	Hypoxidaceae	Herb	Wild	Rhizome	Stir-fry until dry after soaking with wine, then decoction or medicinal liquor for treating impotence, aconuresis. Pounded fresh part applied on the affected area, treating for carbuncle, scrofula	451225130309002
*Curcuma longa* L.	Jianghuang姜黄	–	Zingiberaceae	Herb	both	Tuber	Slicinged and heated applied on the affected area for dissipate blood stasis, dredging collaterals	451225130430037
*Curcuma phaeocaulis* Valeton	Eshu莪术	–	Zingiberaceae	Herb	both	Tuber	Decoction; medicinal bath for dissipate blood stasis, dysmenorrhea	451225130501009
*Cyclea hypoglauca* (Schauer) Diels	Fenyelunhuanteng粉叶轮环藤	ça:u^1^phəp^7^	Menispermaceae	Liana	Wild	Root, Stem, Leaf	Root: Decoction; taken orally for soothe throats, suppressing cough. Stem: Decoction; taken orally for expectorant. Leaf: Decoction; taken orally for sore throat, abdominal pain	451225130310018
*Cynanchum amplexicaule* (Sieb. et Zucc.) Hemsl. var. *castaneum* Makino	Zihuahezhangxiao紫花合掌消	–	Apocynaceae	Herb	both	Whole plant	Decoction; taken orally for cool blood detoxifcation, hepatitis	451225130424025
*Cynanchum atratum* Bunge	Baiwei白薇	–	Apocynaceae	Herb	both	Whole plant	Pounded fresh part applied on the affected area, treating for skin disease	451225130523002
*Cynodon dactylon* (L.) Pers.	Gouyagen狗牙根	khɣət^7^tjen^5^hɣɔk^8^	Poaceae	Herb	Wild	Whole plant	Decoction; taken orally for rheumatism, hemiplegia, over-strained hemoptysis. Pounded fresh part applied on the affected area, treating for traumatic injury, bleeding wound, carbuncle	451225130610024
*Cyperus rotundus* L.	Xiangfuzi香附子	hɣɔk^8^ti^6^cəu^3^	Cyperaceae	Herb	Wild	Rhizome	Decoction; medicinal liquor; taken orally for clearing and activating the channels and collaterals, rheumatism, ostealgia, stomachache, asthma in children	451225130606020
*Daemonorops jenkinsiana* (Griffith) Martius	Huangteng黄藤	ça:u^1^ŋa:n^3^	Arecaceae	Liana	Wild	Stem or root	Decoction; taken orally for food-poisoning, constipation, diarrhea, infectious hepatitis, carbuncle, sore throat	451225130311001
*Damnacanthus indicus* C. F. Gaertn.	Huci虎刺	–	Rubiaceae	Shrub	Wild	Stem and leaf	Decoction; taken orally for treating stone, diuresis, nephropathy	451225121230021
*Datura metel* L.	Baimantuoluo白曼陀罗	ma:n^4^tho^6^lo^5^	Solanaceae	Herb	Home garden	Flower, Leaf	Pounded fresh part applied on the affected area, treating for alopecia. Decoction; medicinal bath, treating for cough with asthma, arthralgia, inchacao, rectal prolapse	451225130523001
*Davallia divaricata* Dutch et Tutch.	Dayegusuibu大叶骨碎补	–	Davalliaceae	Herb	Wild	Rhizome	Medicinal liquor; taken orally or applied on the affected area, for treating rheumatism, strengthening the bones and muscles, traumatic injury	451225130307006
*Dendrobium nobile* Lindl.	Shihu石斛	hɣɔk^8^ŋa:n^3^	Orchidaceae	Herb	Wild	Stem	Decoction; taken orally for febrile diseases, asthenia fever after illness	451225130427039
*Desmodium gangeticum* (L.) DC.	Dayeshanmahuang大叶山蚂蝗	–	Fabaceae	Shrub	Wild	Whole plant	Decoction; taken orally for diuresis	451225121230019
*Desmodium racemosum* (Thunb.) DC.	Shanmahuang山蚂蝗	pɣa^1^miŋ^2^	Fabaceae	Shrub	Wild	Whole plant	Decoction; taken orally for stomachache, infantile malnutrition	451225131109003
*Desmodium multiflorum* DC.	Dongmahuang饿蚂蝗	–	Fabaceae	Shrub	Wild	Root	Decoction; taken orally for clearing away heat and toxic materials, anti-itch, infantile malnutrition	451225130726004
*Dichondra repens* Forst.	Matijin马蹄金	ma^1^luk^7^	Convolvulaceae	Herb	Wild	Whole plant	Decoction; taken orally for throat inflammation, enteritis, liver ascites, jaundice, costalgia, urinary urgency, dysuria, irregular menses. Pounded fresh part applied on the affected area, treating for bleeding wound	451225130610014
*Dicliptera chinensis* (L.) Juss.	Gougancai狗肝菜	ma^1^tap^7^ŋwa^1^	Acanthaceae	Herb	Wild	Whole plant	Decoction; taken orally or medicinal bath for dizziness, tinnitus, bacillary dysentery hemafecia, dysuria, pyretic stranguria, measles	451225130606001
*Dimocarpus longan* Lour.	Longyan龙眼	ȵøn^2^sik^8^	Sapindaceae	Tree	Home garden	Aril	Taken orally directly for weakness of spleen and stomach, anorexia, diarrhea, insomnia dreaminess, palpitation, postpartum hypogalactia	451225130101009
*Dioscorea bulbifcra* L.	Huangdong黄独	kɣa^2^ŋa:n^3^la:k^8^	Dioscoreaceae	Liana	Wild	Tuber	Decoction; taken orally for antral gastritis, enteritis, thyroid disease, cough with lung heat, pudendal ulcer	451225130430035
*Dioscorea cirrhosa* Lour.	Shuliang薯莨	–	Dioscoreaceae	Liana	Wild	Tuber	Stir-fry with rice; taken orally for fever in children	451225130101027, 451225130430011
*Dioscorea esquirolii* Prain et Burkill	Qiyeshuyu七叶薯蓣	–	Dioscoreaceae	Liana	Wild	Rhizome	Decoction; medicinal bath, treating for herpes, hyperthyreosis	451225130312023
*Diospyros kaki* Thunb.	Shi柿	ca:u^1^ma^3^kai^5^	Ebenaceae	Tree	both	Persistent calyx	Decoction; taken orally for vomiting, relieve hiccup	451225130421035, 451225130428004
*Drynaria roosii* Nakaike	Hujue槲蕨	çiŋ^1^mu^6^lau^2^	Polypodiaceae	Herb	Wild	Rhizome	Decocted with water, slicing, drying, medicinal liquor; taken orally for treating kidney deficiency, backache, rheumatoid arthritis, toothache, tinnitus, traumatic injury, bone injury, appendicitis, pelada, heloma	451225130311014, 451225130421012
*Dryopteris championii* (Benth.) C. Chr.	Kuolinlinmaojue阔鳞鳞毛蕨	kon^5^tsɔŋ^1^	Dryopteridaceae	Herb	Wild	Whole plant	Decoction; taken orally for anemopyretic cold ecchymosis, internal hemorrhage, leukorrhea, enteric verminosis	451225130421053
*Duchesnea indica* (Andr.) Focke.	Shemei蛇莓	təm^6^twi^2^	Rosaceae	Herb	Wild	Whole plant	Decoction; taken orally for fever, cough, spitting blood, angina, diarrhea. Pounded fresh part applied on the affected area, treating for abscessfuruncle, snake bite, scald.	451225130311059, 451225130424009
*Dysosma versipellis* (Hance) M. Cheng	Bajiaolian八角莲	–	Berberidaceae	Herb	Wild	Whole plant	Pounded fresh part applied on the affected area, treating for poisonous insect bite	451225130612002
*Dysphania ambrosioides* (L.) Mosyakin et Clemants	Tujingjie土荆芥	ma^1^ȵin^1^	Amaranthaceae	Herb	Wild	Whole plant	Medicinal bath or pounded fresh part applied on the affected area, treating for treating for rheumatism painful swelling, eczema, poisonous insect bite	451225130607023
*Echinochloa crus-galli* (L.) P. Beauv.	Bai稗	–	Poaceae	Herb	Wild	Whole plant	Decoction; taken orally for diuresis detumescence, quiet the spirit	451225130718016
*Eclipta Prostrata* L.	Lichang鳢肠	hɣɔk^8^ma^1^ha:n^5^	Asteraceae	Herb	Wild	Whole plant	Decoction; taken orally or medicinal bath for treating internal hemorrhage, premature graying hair, diphtheritis, turbidity, leukorrhea, pudendal eczema. Pounded fresh part applied on the affected area, treating for bleeding wound, snake bite	451225130421003, 451225130501038
*Elaeagnus glabra* Thunb.	Manhutuizi蔓胡颓子	–	Elaeagnaceae	Liana	Wild	Leaf, Fruit, Root	Leaf: Decoction; taken orally for calm panting and suppress cough. Fruit: Taken orally directly for anti-diarrhea	451225131108045
*Elephantopus scaber* L.	Didancao地胆草	hɣɔk^8^tsja:k^7^ta:ŋ^1^	Asteraceae	Herb	Wild	Whole plant	Decoction; taken orally or medicinal bath for treating gastritis, dental ulcer, pharyngitis, inchacao edema, urinary frequency, urinary urgency, furuncle	451225130806001
*Eleusine indica* (L.) Gaertn.	Niujincao牛筋草	tən^2^cen^1^hɣɔk^8^	Poaceae	Herb	Wild	Whole plant	Decoction; taken orally for fever, damp and hot jaundice, abdominal distention, lumbar muscle injury	451225130610023
*Eleutherococcus nodiflorus* (Dunn) S. Y. Hu	Xizhuwujia细柱五加	ŋɔ^4^ca^1^ŋɣa^2^	Araliaceae	Shrub	Wild	Root bark	Medicinal liquor; taken orally for treating rheumatism, cramp	451225121205001
*Eleutherococcus trifoliatus* (L.) S. Y. Hu	Baiha白簕	–	Araliaceae	Shrub	Wild	Whole plant	Root and leaf: Decoction; taken orally for clearing away heat and toxic materials, nephritis, renal tuberculosis, edema; pounded fresh part applied on the affected area, treating for stanching bleeding; Stem: medicinal liquor; taken orally for rheumatism	451225121205030
*Elsholtzia rugulosa* Hemsl.	Baibeixiangru白背香薷	ma^1^mɣa:ŋ^1^	Lamiaceae	Herb	Wild	Whole plant with flower	Decoction; taken orally or medicinal bath for headache fever, abdominal pain, vomit, diarrhea, edema, inchacao	451225130608041
*Embelia parviflora* Wall. ex A. DC.	Dangguiteng当归藤	–	Primulaceae	Liana	Wild	Stem and leaf	Decoction; taken orally for diuresis, edema	451225121204018
*Emilia sonchifolia* DC.	Yidianhong一点红	nə^5^tjem^3^la:n^3^	Asteraceae	Herb	Wild	Whole plant	Decoction; taken orally or medicinal bath for urinary tract infection, kidney deficiency, sore throat, cough, urinary urgency, furuncle, herpes, eczema	451225130312002
*Epimedium sagittatum* (Sieb. et Zucc.) Maxim.	Sanzhijiuyecao三枝九叶草	hɣɔk^8^ta:n^1^ŋa^5^cəu^3^fa^5^	Berberidaceae	Herb	Wild	Stem, Leaf	Medicinal liquor or stewed with bone and drunk the soup, treating for impotence, dripping discharge of urine, soreness and weakness of waist and knees, rheumatoid arthritis	451225121231009
*Equisetum diffusum* D. Don	Pisanmuzei披散木贼	–	Equisetaceae	Herb	Wild	Whole plant	Pounded fresh part applied on the affected area, for anti-inflammatory, detumescence. Decoction; taken orally for nephritis, diuresis stranguria, renomegaly, clearing heat and improving eyesight	451225130721013
*Equisetum hiemale* L.	Muzei木贼	hɣɔk^8^pət^7^tha:p^7^	Equisetaceae	Herb	Wild	Whole plant	Decoction; taken orally for conjunctivitis, sore throat, abdominal pain, hemafecia, edema	451225131108023
*Eriobotrya japonica* (Thunb.) Lindl.	Pipa枇杷	pε:k^8^pa^2^fa^5^	Rosaceae	Tree	both	Leaf	Decoction; taken orally for ascites due to cirrhosis, cough with lung heat, hemoptysis, clearing away heat and toxic materials	451225130426034
*Eriocaulon buergerianum* Koern.	Gujingcao谷精草	hɣɔk^8^muŋ^4^la^1^	Eriocaulaceae	Herb	Wild	Inflorescence	Decoction; taken orally for nyctalopia, headache, toothache, pharyngitis, hemorrhinia	451225130428017
*Erycibe obtusifolia* Benth.	Dinggongteng丁公藤	ça:u^1^kɔŋ^1^pɔ^1^	Convolvulaceae	Shrub	Wild	Rhizome	Decoction; taken orally for rheumatism, hemiplegia. Pounded fresh part applied on the affected area, treating for painful swelling from knocks and falls	451225130611027
*Eucalyptus globulus* Labill.	Lanan蓝桉	a:n^5^mai^4^fa^5^lo^4^	Myrtaceae	Tree	both	Leaf	Decoction; taken orally for stomachache, prostatitis, wind-heat type common cold, cough, urinary urgency, dysuria. Pounded fresh part applied on the affected area, treating for furuncle, skin itch, eczema	451225130425026
*Eucalyptus robusta* Sm.	An桉	–	Myrtaceae	Tree	both	Seed	Decoction; taken orally for prostatitis, stomachache	451225130310004
*Eucommia ulmoides* oliv.	Dongzhong杜仲	tshja^3^ti^1^ŋɣa^2^	Eucommiaceae	Tree	both	Bark	Stewed with pig kidney and taken orally directly, treating for kidney deficiency, backache, frequent micturition, hypertension. Pounded fresh part applied on the affected area, treating for breaking of muscle and tendon, bone fracture	451225130426035
*Eulaliopsis binata* (Retz.) C. E. Hubb.	Nijinmao拟金茅	–	Poaceae	Herb	Wild	Whole plant	Decoction; taken orally or medicinal bath for clearing liver and eyesight	451225130607032
*Euonymus fortunei* (Turcz.) Hand.-Mazz.	Fufangteng扶芳藤	ça:u^1^fu^6^səu^3^	Celastraceae	Liana	Wild	Stem, Leaf	Medicinal liquor; taken orally or applied on the affected area, treating for rheumatism, ostealgia, traumatic injury, bone fracture. Pounded fresh part applied on the affected area, treating for bleeding wound,	451225130428013
*Euonymus nitidus* Benth.	Zhonghuaweimao中华卫矛	–	Celastraceae	Tree	Wild	Stem and leaf	Medicinal bath for relieve pain	451225130307032
*Eupatorium fortunei* Turcz.	Peilan佩兰	hɣɔk^8^la:n^6^	Asteraceae	Herb	Wild	Stem, Leaf	Decoction; taken orally for acute gastritis and enteritis, blood blight	451225131109021
*Eupatorium lindleyanum* DC.	Linzelan林泽兰	thjen^1^mɛ^1^hɣam^5^	Asteraceae	Herb	Wild	Whole plant	Decoction; taken orally for treating wind-heat type common cold, swelling and aching of gum, cough due to lung heat	451225130427017
*Euphorbia esula* L.	Rujiangdaji乳浆大戟	–	Euphorbiaceae	Herb	Wild	Whole plant	Decoction; medicinal bath for degerming; put it on the bed, treating for chills, fever	451225130306004
*Euphorbia helioscopia* L.	Zeqi泽漆	na:u^3^pa^3^ta:n^5^	Euphorbiaceae	Herb	Wild	Whole plant	Decoction; taken orally or pounded fresh part applied on the affected area, treating for edematous asthma, malaria, bacillary dysentery, scrofula, kerion, osteomyelitis	451225130426030
*Euphorbia hirta* L.	Feiyangcao飞扬草	nɛ^6^hɣo^5^hɣɔk^8^lo^4^	Euphorbiaceae	Herb	Wild	Whole plant	Decoction; taken orally for diarrhea, hematuria, dysuria, herpes eczema	451225121206004
*Euphorbia humifusa* Willd. ex Schltdl.	Dijin地锦	–	Euphorbiaceae	Herb	Wild	Whole plant	Pounded fresh part applied on the affected area, treating for snake bite	451225130306004
*Euphorbia hypericifolia* L.	Tongnaicao通奶草	–	Euphorbiaceae	Herb	Wild	Whole plant	Taken orally directly for diarrhea	451225130420011
*Euphorbia thymifolia* L.	Qiangencao千根草	nɛ^6^hɣo^5^hɣɔk^8^niŋ^5^	Euphorbiaceae	Herb	Wild	Whole plant	Taken orally directly for diarrhea, hemafecia. Pounded fresh part applied on the affected area, treating for eczema, kerion, pruritus	451225140420070
*Euryale ferox* Salisb.	Qian芡	kɣo^3^ci^1^ja^4^	Nymphaeaceae	Herb	Home garden	Fruit	Taken orally directly for enuresis, spermatorrhea, leukorrhea, diarrhea	451225140412008
*Evodia lepta* (Spreng.) Merr.	Sanyaku三桠苦	–	Rutaceae	Tree	Wild	Root, Leaf	Decoction; taken orally or medicinal bath for clearing away heat and toxic materials, anti-itch	451225131109030
*Fagopyrum dibotrys* (D. Don) H. Hara	Jinqiaomai金荞麦	–	Polygonaceae	Herb	both	Whole plant	Pounded fresh part applied on the affected area, treating for mammitis before suppuration	451225130519008
*Ficus carica* L.	Wuhuaguo无花果	khu^5^mɛ^2^hwa^1^hwi^1^	Moraceae	Shrub	both	Receptacle	Decoction; taken orally for diarrhea, constipation, piles, sore throat, cough with lung heat	451225130430049
*Ficus hirta* Vahl	Cuyerong粗叶榕	ŋɔ^4^nja^2^la:k^8^mɔ^6^tɔ^2^	Moraceae	Shrub	Wild	Root	Decoction; taken orally for stomachache, cough, abdominal distension, edema, leukorrhea, rheumatoid arthritis, lumbago	451225130307034
*Ficus microcarpa* L. f.	Rongshu榕树	–	Moraceae	Tree	Wild	Root, Aerial root	Root: medicinal liquor; taken orally for treating raumatic injury, hyperosteogeny, catagma. Aerial root: Decoction with old bamboo and drunk the soup, treating for hemiplegia	451225130430036
*Ficus sarmentosa* Buch.-Ham. ex J. E. Sm. var. *lacrymans* (Levl. Vant.) Corner	Baoyepatengrong薄叶爬藤榕	–	Moraceae	Liana	Wild	Stem and leaf	Decoction; medicinal bath for numbness of bone, rheumatism	451225130423027
*Ficus tikoua* Bur.	Diguo地果	ti^6^ɔŋ^5^	Moraceae	Liana	Wild	Stem, Leaf	Decoction; taken orally for anemopyretic cold, edema, jaundice, rheumatism, piles, amenorrhea, leukorrhea, indigestion, traumatic injury, treating for abdominal paindiarrhea, diarrhea, dizziness due to blood deficiency, leukorrhea, hemorrhinia	451225130423009
*Ficus tinctoria* G. Forst. subsp. *gibbosa* (Blume) Corner	Xieyerong斜叶榕	–	Moraceae	Tree	Wild	Stem and leaf	Decoction; medicinal bath for clearing away heat and toxic materials	451225121205032, 451225130519013, 451225130519028
*Ficus pumila* L.	Bili薜荔	–	Moraceae	Shrub	Wild	Stem and leaf	Decoction with the root of Melastoma malabathricum; medicinal bath for foot pain	451225121231023, 451225130311072
*Flemingia macrophylla* (Willd.) Kuntze ex Prain	Dayeqianjinba大叶千斤拔	–	Fabaceae	Shrub	Wild	Stem and leaf	Decoction; taken orally or medicinal bath, treating for caligo of old people	451225130427015
*Flemingia prostrata* Roxb. f. ex Roxb.	Qianjinba千斤拔	–	Fabaceae	Shrub	Wild	Root	Medicinal liquor; taken orally for treating rheumatism, arthritis, traumatic injury, relaxing tendons and strengthening bones, waist-leg weakness	451225130606029
*Flueggea virosa* (Roxb. ex Willd.) Voigt	Baifanshu白饭树	–	Phyllanthaceae	Shrub	Wild	Stem and leaf	Decoction; medicinal bath for eczema, anti-itch	451225130519010, 451225130606029
*Foeniculum vulgare* Mill.	Huixiang茴香	ma^1^mɣa:ŋ^1^niŋ^5^	Apiaceae	Herb	Home garden	Fruit	Decoction; taken orally for heart and chest pain, abdominal distension, abdominal pain	451225130430031
*Galium aparine* L. var. *echinospermum* (Wallr.) Farw.	Lalateng拉拉藤	hɣɔk^8^pak^7^ta:n^5^	Rubiaceae	Herb	Wild	Whole plant	Decoction; taken orally for treating turbidity, hematuria. Pounded fresh part applied on the affected area, treating for traumatic injury, abscess	451225131108001
*Gardenia jasminoides* J. Ellis	Zhizi栀子	lak^8^mwɔ^2^	Rubiaceae	Shrub	Wild	Fruit	Decoction; taken orally for jaundice with damp-heat pathogen. Incinerated; taken orally with water for treating internal hemorrhage. Pounded fresh part applied on the affected area, treating for sore, oliguria with reddish urine, painful swelling	451225130422008
*Gelsemium elegans* (Gardn. et Champ.) Benth.	Gouwen钩吻	–	Gelsemiaceae	Liana	Wild	Stem and leaf	Frying into carbon shape, decoction; taken orally for treating cancer	451225121204028,
*Geum japonicum* Thunb. var. *chinense* F. Bolle	Roumaolubianqing柔毛路边青	tshjøn^5^məm^6^mai^4^	Rosaceae	Herb	Wild	Whole plant	Decoction; taken orally or medicinal bath for intestinal carbuncle, diarrheabacillary dysentery, toothache, traumatic injury, pudendal pruritus, skin eczema	451225131108027
*Ginkgo biloba* L.	Yinxing银杏	la:k^8^ho^3^pa:k^8^	Ginkgoaceae	Tree	Home garden	Seed	Decoction; taken orally for cough, asthma, nocturnal emission, turbid urine	451225131108049
*Glechoma longituba* (Nakai) Kuprian.	Huoxuedan活血丹	hɣɔk^8^tjen^2^ljen^6^	Lamiaceae	Herb	Wild	Whole plant	Taken orally directly for commom cold, fever, cough, heatstroke, eruptive disease. Pounded fresh part applied on the affected area, cool the blood, dispelling wind detumescence, painful swelling from knocks and falls	451225130309028
*Gleditsia sinensis* Lam.	Zaojia皂荚	thjem^1^teŋ^1^	Fabaceae	Tree	Wild	Thorn	Powdered; applied on the affected area, treating for abscess, sore, kerion, enteritis	451225130308006
*Glochidion eriocarpum* Champ. ex Benth.	Maoguosuanpanzi毛果算盘子	–	Phyllanthaceae	Shrub	Wild	Whole plant	Decoction; taken orally for nephritis, edema	451225130421057, 451225130430045, 451225130421057
*Glochidion puberum* (L.) Hutch.	Suanpanzi算盘子	ton^5^pon^2^.la:k^8^	Phyllanthaceae	Shrub	Wild	Fruit, Stem and leaf	Taken orally directly for malaria, hernia, turbidity, backache. Decoction; medicinal bath for insecticidal anti-itch	451225130608029
*Gnetum parvifolium* (Warb.) Chun	Xiaoyemaimateng小叶买麻藤	–	Gnetaceae	Liana	Wild	Stem	Medicinal liquor; taken orally for rheumatism, activating blood circulation to dissipate blood stasis	451225130310009
*Gomphrena globosa* L.	Qianrihong千日红	thjen^1^fan^1^la:n^3^	Amaranthaceae	Herb	Home garden	Inflorescence or Whole plant	Decoction; medicinal bath for headache, giddiness. Decoction; taken orally for cough and asthma	451225130501040
*Gonostegia hirta* (Bl.) Miq.	Nuomituan糯米团	hu^3^kɣœ^3^ça:u^1^	Urticaceae	Herb	Wild	Whole plant	Decoction; taken orally for diarrhea, leukorrhea, infantile malnutrition, spitting blood. Pounded fresh part applied on the affected area, treating for furuncle, abscess, scrofula, bleeding wound	451225130427019
*Gossypium herbaceum* L.	Caomian草棉	mjεn^2^hwa^1^ta:ŋ^1^	Malvaceae	Herb	Home garden	Whole plant	Decoction; taken orally for weakness cough with asthma, hernia, metrorrhagia and metrostaxis, uterine prolapse	451225130501004
*Gynostemma pentaphyllum* (Thunb.) Makino	Jiaogulan绞股蓝	thət^7^fa^5^mwɔ^5^	Cucurbitaceae	Liana	both	Whole plant	Decoction; taken orally for relieve fever, anti-inflammator, chronic tracheitis, cough and asthma, stomachache, insomnia, headache	451225131109006
*Gynura japonica* (Thunb.) Juel	Jusanqi菊三七	–	Asteraceae	Herb	Wild	Stem and leaf	Pounded fresh part applied on the affected area, treating for traumatic injury, piles	451225130608031
*Gynura bicolor* (Roxb. ex Willd.) DC.	Hongfengcai红凤菜	–	Asteraceae	Herb	Wild	Whole plant	Decoction; taken orally for aid digestion, hypertension	451225130608021
*Hedyotis diffusa* Willd.	Baihuasheshecao白花蛇舌草	hɣɔk^8^ma^2^twi^2^	Rubiaceae	Herb	Wild	Whole plant	Decoction; taken orally for cough with lung heat, sore throat, jaundice, pelvic inflammation. Pounded fresh part applied on the affected area, treating for carbuncle, snake bite	451225130427036
*Helianthus annuus* L.	Xiangrikui向日葵	la:k^8^thəu^5^fan^1^	Asteraceae	Herb	Home garden	Seed, Receptacle	Seed: taken orally directly for treating constipation, bloody dysentery, hemafecia, measles, furuncle. Receptacle: Decoction; taken orally for tinnitus, dizziness, hypertension, dysmenorrhea, constipation	451225121205003
*Helicteres angustifolia* L.	Shanzhima山芝麻	–	Malvaceae	Shrub	Wild	Whole plant	Decoction; taken orally for clearing away heat and toxic materials, detumescence anti-itch, poor urination, removing stasis	451225121205014
*Hemerocallis fulva* L.	Xuancao萱草	ŋa:n^3^hwa^1^ma^1^ta:ŋ^1^	Xanthorrhoeaceae	Herb	both	Root	Decoction; taken orally for edema, dysuria, turbidity, leukorrhea, jaundice, hemafecia, metrorrhagia and metrostaxis, mammary abscess	451225130729014
*Hibiscus mutabilis* L.	Mufurong木芙蓉	mai^4^fu^6^juŋ^6^	Malvaceae	Shrub	Wild	Flower, Leaf, Root	Decoction; taken orally for cough with lung heat, infantile convulsion, leukorrhagia. Pounded fresh part applied on the affected area, treating for furuncle, scald	451225121206003
*Hibiscus sabdariffa* L.	Meiguiqie玫瑰茄	–	Malvaceae	Herb	Home garden	Root	Pounded fresh part applied on the affected area, treating for acute appendicitis	451225121230028
*Hibiscus sgriacus* L.	Mujin木槿	mai^4^cen^1^ŋɣa^2^	Malvaceae	Shrub	Home garden	Bark or Root bark	Decoction; taken orally for diarrhea, hemoptysis, rectal prolapse, piles, eczema, stubborn dermatitis	451225130519029, 451225130722009
*Hordeum vulgare* L.	Damai大麦	mε:k^8^ŋa^2^	Poaceae	Herb	Wild	Fruit	Decoction; taken orally for treating dyspeptic retention, abdominal distention, poor appetite, vomit diarrhea	451225121230036
*Houttuynia cordata* Thunb.	Jicai蕺菜	ma^1^wat^7^	Saururaceae	Herb	Wild	Whole plant	Decoction; taken orally for gynecological disease, tracheitis in children, bronchitis, pneumonia, stone, dermatitis	451225130425034
*Hydrocotyle sibthorpioides* Lam.	Tianhusui天胡荽	–	Apiaceae	Herb	Wild	Whole plant	Decoction; taken orally for jaundice hepatitis, lithangiuria	451225121231004
*Hypericum japonicum* Thunb.	Didongcao地耳草	ça:ŋ^1^tsən^2^	Clusiaceae	Herb	Wild	Whole plant	Decoction; taken orally for gynecological inflammation, liver ascites, damp and hot jaundice, intestinal carbuncle. Pounded fresh part applied on the affected area, treating for snake bite, furuncle abscess	451225130423003, 451225130427018, 451225130610029
*Hypericum sampsonii* Hance	Yuanbaocao元宝草	hɣɔk^8^ȵen^6^pɔ^1^	Clusiaceae	Herb	Wild	Whole plant	Decoction; taken orally for internal hemorrhage, irregular menses, dysmenorrhea. Pounded fresh part applied on the affected area, treating for bleeding wound	451225130426017, 451225130518027
*Ilex asprella* (Hook. et Arn.)_champ. ex Benth.	Chengxingshu秤星树	mai^4^ja^4^həu^1^	Aquifoliaceae	Tree	Wild	Stem and leaf	Decoction; taken orally for bitter taste, common cold, eruptive disease, abscess of lung, hemoptysis, sore throat, gonorrhea. Pounded fresh part applied on the affected area, treating for carbuncle toxin, traumatic injury	451225121231014
*Ilex rotunda* Thunb.	Tiedongqing铁冬青	cəu^5^lai^3^çen^1^	Aquifoliaceae	Tree	Wild	Bark	Decoction; taken orally for fever, sore throat, damp and hot diarrhea, stomachache, hemoptysis, spitting blood, hemafecia, hematuria. Powdered; applied on the affected area, treating for traumatic injury	451225130101003
*Illicium verum* Hook. f.	Bajiao八角	–	Schisandraceae	Tree	both	Whole plant	Pounded fresh part applied on the affected area, treating for facial skin disease	451225130430031
*Impatiens balsamina* L.	Fengxianhua凤仙花	–	Balsaminaceae	Herb	Wild	Whole plant, Seed	Decoction; medicinal bath for rheumatoid arthritis, contracture of bones and muscles, inchacao, tinea sores	451225130519022
*Imperata cylindrica* (L.) Raeusch.	Baimao白茅	juŋ^3^nɔ^3^	Poaceae	Herb	Wild	Rhizome	Decoction; taken orally for edema, jaundice, pancreatitis, mastitis, internal hemorrhage, edema, damp and hot jaundice	451225130101017
*Ipomoea nil* (Linnaeus ) Roth	Qianniu牵牛	chen^1^tən^2^la:k^8^	Convolvulaceae	Herb	Wild	Seed	Taken orally directly for treating edema, inchacao, constipation	451225121206008, 451225130718012
*Isatis tinctoria* L.	Songlan崧蓝	lo^4^sən^3^fa^5^	Brassicaceae	Herb	Home garden	Root	Decoction; taken orally for influenza, epidemic encephalitis B, sore throat, mumps, red eyes, pneumonia, erysipelas, herpes	451225130102011
*Ixeris polycephala* Cass.	Kumaicai苦荬菜	ma^1^kam^1^	Asteraceae	Herb	both	Whole plant	Decoction; taken orally for abscess of lung, mammary abscess, bloody stranguria, furuncle. Pounded fresh part applied on the affected area, treating for traumatic injury	451225130424008
*Jasminum nudiflorum* Lindl.	Yingchunhua迎春花	jin^6^tshən^1^hwa^1^	Oleaceae	Shrub	Home garden	Flower	Decoction; taken orally or medicinal bath for treating fever headache, painful voidings of hot urine, carbuncle eczema	451225130307012
*Jasminum sambac* (L.) Aiton	Molihua茉莉花	–	Oleaceae	Shrub	both	Root	Medicinal liquor; taken orally for treating rheumatism	451225130307014
*Juglans regia* L.	Hutao胡桃	hwi^1^tɔ^2^	Juglandaceae	Tree	Home garden	Seed	Taken orally directly for kidney deficiency, dyspnea with cough, backache, impotence, spermatorrhea, frequent micturition, dry feces	451225130307017
*Juncus effusus* L.	Dengxincao灯心草	hɣɔk^8^fi^1^taŋ^1^	Juncaceae	Herb	Wild	Whole plant	Decoction; taken orally for insomnia, prostatitis, lithangiuria	451225130422017, 451225130501023
*Justicia adhatoda* L.	Yazuihua鸭嘴花	–	Acanthaceae	Shrub	Wild	Whole plant	Pounded fresh part applied on the affected area, treating for protrusion of lumbar intervertebral disc, snake bite, traumatic injury	451225130307025
*Justicia ventricosa* Wall. ex Sims.	Heiyexiaobogu黑叶小驳骨	–	Acanthaceae	Herb	Wild	Whole plant	Pounded fresh part applied on the affected area, treating for traumatic injury, hyperosteogeny, protrusion of lumbar intervertebral disc, scald	451225130607011
*Kadsura coccinea* (Lem.) A. C. Smith	Heilaohu黑老虎	ça:u^1^kon^3^kɔk^8^	Schisandraceae	Liana	Wild	Stem, Leaf	Medicinal liquor; taken orally for liver ascites, rheumatism, ostealgia. Pounded fresh part applied on the affected area, treating for traumatic injury, bone fracture, furuncle, wound infection	451225130307040
*Kadsura longipedunculata* Finet et Gagnep.	Nanwuweizi南五味子	–	Schisandraceae	Liana	Wild	Root, Stem, Fruit	Root and stem: Decoction; taken orally for gastritis. Fruit: medicinal liquor; taken orally for treating rheumatism, stomachache	451225130308007
*Kalimeris indica* (L.) Sch. Bip.	Malan马兰	–	Asteraceae	Herb	Wild	Whole plant	Pounded fresh part applied on the affected area, treating for removing blood stasis, clearing away heat and toxic materials	451225130309013
*Kummerowia striata* (Thunb.) Schindl.	Jiyancao鸡眼草	hɣɔk^8^ci^1^la^1^	Fabaceae	Herb	Wild	Whole plant	Decoction; taken orally for cold and fever, vomiting and diarrhea, malaria, diarrhea, infectious hepatitis	451225130608028
*Kyllinga polyphylla* Kunth	Shuiwugong水蜈蚣	hɣɔk^8^nəm^4^cε^3^khɣap^7^	Cyperaceae	Herb	Wild	Whole plant or Root	Decoction; taken orally for fever, cough, diarrhea bacillary dysentery. Medicinal liquor; taken orally for traumatic injury, rheumatism	451225130309030
*Kyllinga nemoralis* (J. R. Forster & G. Forster) Dandy ex Hutchinson & Dalziel	Dansuishuiwugong单穗水蜈蚣	–	Cyperaceae	Herb	Wild	Whole plant	Decoction; taken orally for commom cold, cough, clearing and activating the channels and collaterals, pneamopathy, renomegaly	451225130519004
*Lablab purpureus* (L.) Sweet	Biandong扁豆	tau^6^pɔp^7^	Fabaceae	Liana	Home garden	Seed	Decoction; taken orally for diarrhea, vomit, bacillary dysentery	451225130309043
*Lantana camara* L.	Mayingdan马缨丹	ŋɔ^4^sak^7^hwa^1^	Verbenaceae	Shrub	Wild	Stem and leaf	Branch and leaf: Pounded fresh part applied on the affected area, treating for itchy skin, eczema, traumatic injury, painful swelling. Root: Decoction; taken orally for treating kidney stone	451225130429022
*Laportea violacea* Gagnep.	Putaoyeaima葡萄叶艾麻	–	Urticaceae	Herb	Wild	Root	Stewed with pig spleen and drunk the soup, treating for ascites due to cirrhosis	451225130310042
*Lemmaphyllum microphyllum* C. Presl var. *obovatum* (Harr.) C. Chr.	Daoluanyefushijue倒卵叶伏石蕨	–	Polypodiaceae	Herb	Wild	Whole plant	Decoction; taken orally for infantile malnutrition	451225130311011
*Leonurus japonicus* Houtt.	Yimucao益母草	mau^6^mai^4^hɣɔk^8^	Lamiaceae	Herb	Wild	Whole plant	Decoction; taken orally for irregular menses, amenorrhea, dysmenorrhea, postpartum blood stasis, abdominal pain, persistent lochia. Pounded fresh part applied on the affected area, treating for edema, abscess, pruritus, traumatic injury	451225130426002, 451225130518028, 451225130606006
*Lespedeza cuneata* (Dum.-Cours.) G. Don	Jieyetiesaozhou截叶铁扫帚	mu^2^kwa:n^1^tɔ^1^	Fabaceae	Shrub	Wild	Whole plant	Decoction; medicinal bath for dissipate blood stasis detumescence	451225130311017
*Ligustrum lucidum* Ait.	Nvzhen女贞	tsɔŋ^1^tsən^5^la:k^8^	Oleaceae	Tree	both	Fruit	Decoction; taken orally for liver ascites, soreness and weakness of waist and knees, tinnitus and dizziness	451225130718011
*Ligustrum quihoui* Carr.	Xiaoyenvzhen小叶女贞	–	Oleaceae	Shrub	Wild	Stem and leaf	Medicinal bath, treating for clearing away heat and toxic materials	451225130311048
*Lilium brownii* F. E. Br. ex Miellez	Yebaihe野百合	–	Liliaceae	Herb	Wild	Bulb	Stewed with meat and taken orally directly for cough with lung heat, expectoration, dysphoria, palpitation, insomnia	451225130518030, 451225130519050
*Lindera aggregata* (Sims) Kosterm.	Wuyao乌药	u^1^kɣa^2^	Lauraceae	Tree	Wild	Root	Decoction; taken orally for abdominal distention, abdominal pain, urinary frequency	451225130312003
*Liquidambar formosana* Hance	Fengxiangshu枫香树	mai^4^hɣəu^1^la:k^8^	Altingiaceae	Tree	Wild	Fruit	Medicinal liquor; taken orally for rheumatism, removing blood, spasm of hand and foot. Decoction; taken orally for stomachache, edema, carbuncle, anal fistula, eczema	451225130312012
*Liriope spicata* (Thunb.) Lour.	Shanmaidong山麦冬	–	Asparagaceae	Herb	Wild	Whole plant	Decoction; taken orally for hepatopathy. Stewed with meat and drank the soup for treating jaundice hepatitis	451225130312016
*Litchi chinensis* Sonn.	Lizhi荔枝	li^6^tsi^1^la:k^8^	Sapindaceae	Tree	Home garden	Aril, Seed	Seed: taken orally directly for epigastralgia, hernia, dysmenorrhea, eliminating stagnation. Fruit: taken orally directly for polydipsia, hiccup	451225130730006
*Litsea cubeba* (Lour.) Per.	Shanjijiao山鸡椒	–	Lauraceae	Tree	Wild	Root, Stem, Leaf	Stewed with meat and drunk the soup, treating for removing wind and dispersing cold, smooth circulation and stop pains	451225130310026, 451225130430046, 451225130519032, 451225130610028
*Litsea pungens* Hemsl.	Mujiangzi木姜子	ja^4^mai^4^tsja:ŋ^5^la:k^8^	Lauraceae	Tree	Wild	Fruit	Decoction; taken orally for anemofrigid cold, abdominal distention, poor appetite. Pounded fresh part applied on the affected area, treating for bleeding wound	451225130421018
*Lobelia chinensis* Lour.	Banbianlian半边莲	mɣa:ŋ^6^pjen^1^ljen^2^	Campanulaceae	Herb	Wild	Whole plant	Decoction; taken orally for jaundice, edema, abdominal distension, diarrhea, diarrhea. Pounded fresh part applied on the affected area, treating for snake bite, furuncle abscess, sprain	451225130501028, 451225130606026
*Lonicera confusa* (Sweet) DC.	Huananrendong华南忍冬	cəm^1^ȵen^2^ça:u^1^	Caprifoliaceae	Liana	both	Stem, Bud	Stem: Medicinal bath, treating for abscess, rheumatism. Flower: Decoction; taken orally for treating for fever, bloody flux, carbuncle, swollen toxin, scrofula, hemorrhoid complicated by anal fistula	451225130422035
*Lonicera hypoglauca* Miq.	Guxianrendong菰腺忍冬	–	Caprifoliaceae	Liana	Both	Whole plant	Decoction; taken orally or applied on the affected area, treating for headache, liver ascites, skin disease	451225130421045, 451225130719005
*Lophatherum gracile* Brongn.	Danzhuye淡竹叶	kwan^1^ta:m^6^fa^5^	Poaceae	Herb	Wild	Whole plant	Decoction; taken orally for tongue and mouth sores, dysuria, cough with lung heat, infantile convulsions, insomnia, uterine bleeding, apoplexy, threatened abortion	451225130422050
*Loranthus* sp.	Sangjishengshuyizhong桑寄生属一种	–	Loranthaceae	Shrub	Wild	Stem and leaf	Decoction; taken orally for treating inchacao, rheumatoid arthritis, postpartum hypogalactia	451225130423005
*Loranthus* sp.	Sangjishengshuyizhong桑寄生属一种	–	Loranthaceae	Shrub	Wild	Whole plant	Decoction; taken orally for cough, cold	451225130423015
*Luffa cylindrica* (L.) Roem.	Sigua丝瓜	thjen^1^la^2^hɣə:n^5^	Cucurbitaceae	Liana	Home garden	Peel	Decoction; taken orally for cough with lung heat, testicle painful swelling, amenorrhoea, promoting lactation	451225130423016
*Lycopodium japonicum* Thunb.	Shisong石松	hɣɔk^8^hɣaŋ^4^cen^1^	Lycopodiaceae	Herb	Wild	Whole plant	Medicinal liquor ; taken orally or applied on the affected area, for treating rheumatoid arthritis, numbness of limbs, edema, traumatic injury	451225130424032
*Lycopus lucidus* Turcz. ex Benth.	Disun地笋	tsek^8^la:m^2^	Lamiaceae	Herb	Wild	Stem, Leaf	Pounded fresh part applied on the affected area, treating for amenorrhea abdominal pain, edema, traumatic injury, carbuncle, swelling and pain	451225130425002
*Lycoris radiata* (L’Hey.) Herb.	Shisuan石蒜	hɣɔ^2^mən^1^	Amaryllidaceae	Herb	Wild	Bulb	Decoction; taken orally for anemofrigid cold, cough. Pounded fresh part applied on the affected area, treating for edema	451225130425021
*Lygodium japonicum* (Thunb.) Sw.	Haijinsha海金沙	–	Lygodiaceae	Herb	Wild	Whole plant	Decoction; taken orally for kidney stone, clearing heat and diuresis, stranguria. Pounded fresh part applied on the affected area for anaesthesia	451225121204033, 451225130311055, 451225130606031
*Lygodium microphyllum* (Cav.) R. Br.	Xiaoyehaijinsha小叶海金沙	–	Lygodiaceae	Herb	Wild	Whole plant	Decoction; taken orally for kidney stone, heat-clearing and diuresis, stranguria	451225130425040
*Lysionotus pauciflorus* Maxim.	Diaoshijutai吊石苣苔	–	Gesneriaceae	Shrub	Wild	Whole plant	Pounded fresh part applied on the affected area, treating for traumatic injury	451225130723003
*Maclura cochinchinensis* (Lour.) Corner	Gouji构棘	ta:ŋ^1^lyn^1^cet^7^	Moraceae	Shrub	Wild	Root	Medicinal liquor; taken orally or applied on the affected area, treating for rheumatoid arthralgia, traumatic injury. Decoction; taken orally for jaundice, turbidity, menostasis, hemoptysis, furuncle abscess	451225131108028
*Magnolia liliflora* Desr.	Xinyi辛夷	çin^5^tshən^6^hwa^1^	Magnoliaceae	Tree	Home garden	Flower	Decoction; taken orally for headache, nasosinusitis	451225130426014
*Mahonia bealei* (Fortune) Carrière	Kuoyeshidagonglao阔叶十大功劳	ŋa:n^3^mai^4^ljen^2^	Berberidaceae	Shrub	Wild	Leaf	Decoction; taken orally for clearing away heat and resolving fire, treating for headache, cough, jaundice	451225130728014
*Mahonia* sp.	Shidagonglaoshu十大功劳属	–	Berberidaceae	Shrub	Wild	Stem	Decoction; taken orally for clearing away heat and reducing fire, internal thermal, pneumonia	451225130427006
*Mallotus paniculatus* (Lam.) Muell. Arg.	Baiqiu白楸	fa^5^ləu^2^pa:k^8^	Euphorbiaceae	Tree	Wild	Root, Leaf	Root: Decoction; taken orally for leukorrhea, infertility. Leaf: pounded fresh part applied on the affected area, treating for bleeding wound, traumatic injury, thrush, bedsore	451225130427007
*Malva verticillata* L. var. *crispa* L.	Dongkui冬葵	tɔŋ^6^thəu^5^fan^1^	Malvaceae	Herb	Home garden	Seed	Decoction; taken orally for constipation, poor urination, insufficient lactation	451225130427013
*Marsilea quadrifolia* L. Sp.	Ping苹	–	Marsileaceae	Herb	Wild	Whole plant	Decoction; taken orally for liver ascites	451225130425012, 451225130519011
*Melastoma dodecandrum* Lour.	Dinie地菍	–	Melastomataceae	Herb	Wild	Whole plant	Decoction; taken orally for hepatopathy	451225130422007
*Melastoma malabathricum* L.	Yemudan野牡丹	–	Melastomataceae	Shrub	Wild	Root	Medicinal bath for painful swelling of feet	451225130422005, 451225130610026, 451225131109025
*Melia azedarach* L.	Lian楝	mai^4^khu^1^ljen^6^ta:ŋ^1^ŋɣa^2^	Meliaceae	Tree	Wild	bark	Decoction; taken orally or medicinal bath for treating for depriving ascarid, enterobiasis, measles, hemorrhoids	451225130611026
*Mentha canadensis* L.	Baohe薄荷	po^6^o^5^	Lamiaceae	Herb	both	Whole plant	Taken orally directly for treating affection of exogenous wind-heat, headache, fever, red eyes, measles	451225130427024
*Millettia pachyloba* Drak*e*	Hainanyadouteng海南崖豆藤	ça:u^1^tɔk^8^məm^6^	Fabaceae	Liana	Wild	Root, Stem, Leaf	Medicinal liquor; taken orally or applied on the affected area, treating for scabies, wet leprosy, rheumatic arthritis	451225130428018
*Mimosa pudica* L.	Hanxiucao含羞草	hɣɔk^8^khɣə:n^5^jɛ^6^	Fabaceae	Herb	Wild	Whole plant	Decoction; taken orally or medicinal bath for gastritis, enteritis, insomnia, infantile malnutrition, herpes zoster	451225130428034
*Miscanthus sinensis* Andersson	Mang芒	–	Poaceae	Herb	Wild	Whole plant	Decoction; taken orally for relieve pain, stanching bleeding, enteritis	451225130310057
*Momordica charantia* L.	Kugua苦瓜	ku^1^li^5^fa^5^	Cucurbitaceae	Liana	Home garden	Leaf	Decoction; taken orally for stomachache, diarrhea. Pounded fresh part applied on the affected area, treating for eczema, prickly heat	451225130718020
*Morinda officinalis* How.	Bajitian巴戟天	hɣɔk^8^ci^1^khɣe^3^	Rubiaceae	Liana	Wild	Root	Powdered; taken orally with water or liquor, treating for impotence, aconuresis, rheumatoid arthritis, soreness and weakness of waist and knees	451225130428047
*Morus alba* L.	Sang桑	saŋ^5^la:k^8^	Moraceae	Shrub	Home garden	Whole plant	Root: Decoction; taken orally for diuresis. Branch: medicinal liquor; taken orally or rinsed the affected area, treating for rheumatism. Leaf: Decoction; taken orally for clear wind-heat. Fruit: taken orally directly or medicinal liquor and taken orally for tonifying liver and kidney	451225130311060, 451225130421060
*Murraya paniculata* (L.) Jack.	Qianlixiang千里香	–	Rutaceae	Shrub	Wild	Root, Stem	Decoction; taken orally for heart disease	451225121231001, 451225130311022
*Musa basjoo* Sieb. et Zucc.	Bajiao芭蕉	fja:k^7^ta:ŋ^1^	Musaceae	Herb	Home garden	Rhizome	Decoction; taken orally or medicinal bath for jaundice, edema, inchacao, bloody stranguria, metrorrhagia, furuncle, erysipelas	451225130429011
*Mussaenda erosa* Champ. ex Benth.	Nanteng楠藤	–	Rubiaceae	Liana	Wild	Stem and leaf	Decoction; taken orally or medicinal bath for clearing away heat and relieving exterior syndrome, infertility	451225130421042, 451225130430005
*Nandina domestica* Thunb.	Nantianzhu南天竹	–	Berberidaceae	Shrub	both	Stem and leaf	Decoction; taken orally for cooling blood	451225130102005, 451225130426025
*Nelumbo nucifera* gaerth.	Lian莲	ŋau^4^la:k^8^təm^1^	Nelumbonaceae	Herb	Home garden	Leaf, Seed, Germ	Leaf: Decoction; taken orally for diarrhea, vertigo, edema, internal hemorrhage. Seed: Taken orally directly for upset, spitting blood, spermatorrhea, swelling and pain of eye	451225130429017
*Neolepisorus fortunei* (T. Moore) Li Wang	Jiangnanxingjue江南星蕨	–	Polypodiaceae	Herb	Wild	Whole plant	Decoction; taken orally for rheumatism	451225121205019
*Nepeta cataria* L.	Jingjie荆芥	mai^4^jin^1^	Lamiaceae	Herb	Wild	Whole plant	Decoction; taken orally for fever commom cold, headache, sore throat, internal hemorrhage, metrorrhagia and metrostaxis, postpartum anemic fainting, abscess, sores, scrofula	451225130430022
*Nervilia plicata* (Andrews) Schltr.	Maoyeyulan毛叶芋兰	həu^1^ljen^2^	Orchidaceae	Herb	Wild	Leaf, Tuber	Decoction; taken orally for tuberculosis, cough with lung heat, hemoptysis. Pounded fresh part applied on the affected area, treating for scrofula, swollen toxin, traumatic injury	451225130430041
*Ocimum basilicum* L.	Luole罗勒	–	Lamiaceae	Herb	Wild	Whole plant, Seed	Pounded fresh part applied on the affected area, treating for dispelling wind detumescence, dissipate blood stasis relieve pain	451225130430048
*Onychium japonicum* (Thunb.) *Kze.*	Yezhiweijinfenjue野雉尾金粉蕨	–	Pteridaceae	Herb	Wild	Whole plant	Decoction; taken orally for calculosis	451225130102002
*Ophiopogon intermedius* D. Don	Jianxingyanjiecao间型沿阶草	mε:k^8^tɔŋ^1^	Asparagaceae	Herb	Wild	Rhizome	Stewed with meat and taken orally directly for treating irritating dry cough, hemoptysis, angina, abscess of lung, diabetes, constipation due to intestinal dryness	451225130501003
*Opuntia dillenii* (Ker Gawl.) Haw.	Xianrenzhang仙人掌	tɔŋ^6^pən^6^tsja:ŋ^3^	Cactaceae	Shrub	both	Root, Stem	Pounded fresh part applied on the affected area, treating for abdominal pain, diarrhea, scald, snake bite	451225130728015
*Oryza sativa* L.	Dao稻	hu^3^kɔk^7^ŋa^2^	Poaceae	Herb	Home garden	Seed-bud	Decoction; taken orally for treating dyspeptic retention, indigestion	451225130501036
*Oxalis corniculata* L.	Zhajiangcao酢浆草	ma^1^khɣəm^3^	Oxalidaceae	Herb	Wild	Whole plant	Decoction; taken orally for diarrhea, gonorrhea, leukorrhea, measles, internal hemorrhage, sore throat, abscess, piles, rectal prolapse. Pounded fresh part applied on the affected area, treating for traumatic injury, scald	451225130721007
*Paederia scandens* (Lour.) Merr.	Jishiteng鸡矢藤	ça:u^1^cɛ^3^ci^1^	Rubiaceae	Liana	Wild	Whole plant	Pounded fresh part applied on the affected area, treating for snake bite, itching	451225130102016
*Paederia scandens* (Lour.) Merr. var. *tomentosa* (Bl.) Hand.-Mazz.	Maojishiteng毛鸡矢藤	ça:u^1^ci^1^cɛ^3^pa:k^8^	Rubiaceae	Liana	Wild	Root or Whole plant	Decoction; taken orally for jaundice, diarrhea, dyspeptic retention, amenorrhea	451225130501037
*Pandanus austrosinensis* T. L. Wu	Ludongcao露兜草	–	Pandanaceae	Herb	Wild	Fruit	Leaf: Decoction; taken orally or medicinal bath for renomegaly, diuresis, sweating, anti-inflammatory. Fruit: Decoction; taken orally for cough, nephritis	451225130310036
*Paris polyphylla* Sm. var. *chinensis* (Franch.) Hara	Qiyeyizhihua七叶一枝花	thət^7^fa^5^ljen^2^	Trilliaceae	Herb	both	Rhizome	Powdered, taken orally or applied on the affected area for abscess furuncle, scrofula, sore throat, chronic tracheitis, infantile convulsion, snake bite	451225130518006
*Passiflora papilio* H. L. Li	Hudieteng蝴蝶藤	–	Passifloraceae	Liana	Wild	Whole plant	Medicinal liquor or decoction; taken orally for rheumatism, paralysis, indigestion	451225130726016
*Patrinia villosa* (Thunb.) Juss.	Pandaozeng攀倒甑	hɣɔk^8^ja:ŋ^6^tsja:ŋ^5^	Caprifoliaceae	Herb	Wild	Whole plant	Decoction; taken orally or medicinal bath for treating intestinal carbuncle, diarrhea, leukorrhea, abdominal pain, red eyes swollen toxin, abscess, hemorrhoids	451225130718007, 451225131108012
*Paulownia fortunei* (Seem) Hemsl.	Baihuapaotong白花泡桐	mai^5^phɔ^5^tɔŋ^2^	Scrophulariaceae	Tree	Wild	Bark	Decoction; taken orally for treating rheumatism, arthritis, edema, toxic heat, scabies	451225130518011
*Pentarhizidium orientale* Hayata	Dongfangjiaguojue东方荚果蕨	–	Onocleaceae	Herb	Wild	Whole plant	Decoction; taken orally for hepatitis, carditis	451225130518012
*Penthorum chinense* Pursh	Chegencai扯根菜	–	Penthoraceae	Herb	Wild	Whole plant	Pounded fresh part applied on the affected area, treating for traumatic injury	451225130518034
*Pericampylus glaucus* (Lam.) Merr.	Xiyuanteng细圆藤	ça:u^1^nam^2^fɔŋ^1^	Menispermaceae	Liana	Wild	Stem or root	Decoction; taken orally for infantile convulsions	451225130423031, 451225130611009
*Perilla frutescens* (L.) Britton	Zisu紫苏	–	Lamiaceae	Herb	Home garden	Stem, Leaf, Seed	Taken orally directly for dissipate wind-cold, relieve stasis and dissipate phlegm, ichthyotoxin, fish poison, turtle poison	451225130519025
*Perilla frutescens* (L.) Britton var. *purpurascens* (Hayata) H. W. Li	Yeshengzisu野生紫苏	–	Lamiaceae	Herb	both	Whole plant	Taken orally directly for cold. Pounded fresh part applied on the affected area, promoting wound healing	451225130518038
*Perilla frutescensc* (L.) Britt. var. *crispa* (Thunb.) Hand-Mazz.	Huihuisu回回苏	lau^5^ma^1^fa^5^	Lamiaceae	Herb	Home garden	Whole plant	Root: Decoction; taken orally for anemofrigid cold, cough, abdominal distention, threatened abortion, fish poison, turtle poison. Seed: Taken orally directly for cough and asthma, constipation due to intestinal dryness. Stem: Decoction; taken orally for threatened abortion, abdominal distension	451225130426018
*Pholidota chinensis* Lindl.	Shixiantao石仙桃	hwi^1^tɔ^2^fa^5^	Orchidaceae	Herb	Wild	Tuber or Whole plant	Decoction; taken orally for cough, hemoptysis, cough with lung heat, nocturnal emission. Pounded fresh part applied on the affected area, treating for scrofula, traumatic injury	451225130101026
*Phragmites australis* (Cav.) Trin. ex Steud.	Luwei芦苇	kɣo^3^ŋɔ^4^	Poaceae	Shrub	Wild	Rhizome	Decoction; taken orally for prostatitis, nephritis, vomiting due to stomach heat, nausea, abscess of lung, oliguria with reddish urine	451225130606041
*Phyllanthus urinaria* L.	Yexiazhu叶下珠	–	Phyllanthaceae	Herb	Wild	Whole plant	Decoction; taken orally or medicinal bath for detumescence improving eyesight, diuresis	451225130611023, 451225130718026, 451225130611023
*Phyllodium pulchellum* (L.) Desv.	Paiqianshu排钱树	pa:i^2^tjen^2^hɣɔk^8^	Fabaceae	Shrub	Wild	Root, Leaf	Decoction; taken orally for clearing away heat and relieving exterior syndrome. Medicinal liquor; taken orally for treating removing blood and dissipate blood stasis	451225130607012
*Physalis angulata* L.	Kuta苦蘵	–	Solanaceae	Herb	Wild	Whole plant	Decoction; taken orally for clearing away heat and toxic materials, expectorants up pressing cough	451225130718031
*Physalis peruviana* L.	Denglongguo灯笼果	tɔŋ^6^kwi^5^pɔm^1^	Solanaceae	Herb	both	Whole plant	Decoction; taken orally for common cold, sore throat, hernia. Pounded fresh part applied on the affected area, treating for poisoned sore	451225130429006
*Phytolacca acinosa* Roxb.	Shanglu商陆	lən^1^ləm^6^tjeu^1^	Phytolaccaceae	Herb	Wild	Root	Decoction; taken orally for edema, antral gastritis, gastric bleeding, constipation, diuresis, abscess	451225130429035, 451225130518026
*Phytolacca americana* L.	Chuixushanglu垂序商陆	–	Phytolaccaceae	Herb	Wild	Root	Decoction; medicinal bath for skin disease	451225130609004
*Pilea cavaleriei* H. Lév.	Shiyoucai石油菜	pi^2^ma^1^mu^5^	Urticaceae	Herb	Wild	Whole plant	Decoction; taken orally for cough due to tuberculosis, cough with lung heat. Pounded fresh part applied on the affected area, treating for scald, sores painful swelling	451225130608032
*Pinellia ternata* (Thunb.) Breit.	Banxia半夏	ma^1^ɣa:k^7^la:k^8^	Araceae	Herb	Wild	Tuber	Pounded fresh part applied on the affected area, treating for furuncle abscess	451225130306013
*Pinus massoniana* Lamb.	Maweisong马尾松	tsuŋ^6^pε:k^7^jəu^2^	Pinaceae	Tree	Wild	Stem tubercle, Leaf	Branchlet tubercle: medicinal liquor, taken orally or rinsed the affected area, treating for rheumatic arthritis, tuberculous arthritis, blood stasis. Leaf: Decoction; taken orally and rinsed for rheumatoid arthritis, traumatic injury, insomnia, edema, eczema, hemorrhoids	451225130610011
*Piper kadsura* (Choisy) Ohwi.	Fengteng风藤	ta^6^pɣa^1^lɔŋ^2^fa^5^lo^4^	Piperaceae	Herb	Wild	Stem	Medicinal liquor; taken orally or applied on the affected area, treating for rheumatoid arthritis, joint pain, vessel contracture syndromes etc., traumatic injury	451225130610012
*Piper nigrum* L.	Hujiao胡椒	hu^2^tjeu^1^	Piperaceae	Herb	Wild	Fruit	Taken orally directly for cold phlegm and dyspepsia, nausea, vomit, diarrhea, cold type dysentery, food-poisoning	451225130610017
*Piper wallichii* (Miq.) Hand.-Mazz.	Shinanteng石南藤	–	Piperaceae	Liana	Wild	Whole plant	Medicinal liquor; taken orally for treating rheumatism, ostealgia, waist-leg weakness, cough and asthma	451225130310071, 451225130425006
*Plantago asiatica* L.	Cheqian车前	tu^3^mu^5^ma^1^	Plantaginaceae	Herb	Wild	Whole plant	Decoction; taken orally for enriching blood, clearing away heat and dampness, diuresis stranguria, hematuria, urinary tract infection, nephritis	451225130309004
*Platycladus orientalis* (L.) Franco	Cebai侧柏	pə^6^fa^5^	Cupressaceae	Tree	both	Stem and leaf	Decoction; taken orally for piles, internal hemorrhage, hemorrhoidal hamorrhage, metrorrhagia and metrostaxis, bacillary dysentery, cough, seborrhoeic dermatitis, alopecia	451225130611007
*Plumbago zeylanica* L.	Baihuadan白花丹	–	Plumbaginaceae	Herb	both	Whole plant	Decoction; medicinal bath, treating for edema, infantile malnutrition	451225121205037, 451225130606038
*Pogostemon cablin* (Blanco) Benth.	Guanghuoxiang广藿香	khɔ^6^mɣa:ŋ^1^	Lamiaceae	Herb	Wild	Whole plant	Taken orally with saline water for abdominal distention, poor appetite, nausea, vomit	451225130611029
*Polygala japonica* Houtt.	Guazijin瓜子金	hɣɔk^8^kwa^1^la:k^8^	Polygalaceae	Herb	Wild	Whole plant or Root	Decoction; taken orally for sore throat, cough with copious phlegm, pertussis cough, abscess, traumatic injury, insomnia	451225130804002
*Polygonatum cyrtonema* Hua	Donghuahuangjing多花黄精	ci^1^ŋa:n^3^ma^1^	Asparagaceae	Herb	Wild	Rhizome	Slicing and decoction; taken orally directly for tuberculosis hemoptysi, weakness, soreness and weakness of waist and knee, rheumatoid arthritis	451225000000000
*Polygonum chinense* L.	Huotanmu火炭母	–	Polygonaceae	Herb	Wild	Whole plant	Decoction; taken orally for relieve pain and inflammation, ulcer	451225130501031
*Polygonum hydropiper* L.	Shuiliao水蓼	–	Polygonaceae	Herb	Wild	Whole plant	Decoction; medicinal bath for killing parasites to relieve itshing, eczema	451225130718008
*Polygonum multiflorum* Thunb.	Heshouwu何首乌	ma^1^tap^7^twi^2^	Polygonaceae	Liana	Wild	Rhizome	Decoction; taken orally for insomnia, profuse sweating, skin eruption, kidney deficiency, premature graying of the hair, dizzy of the head and dim of sight, soreness and weakness of waist and knees, spermatorrhea, chronic hepatitis, abscess, constipation due to intestinal dryness	451225130428007
*Polygonum orientale* L.	Hongliao红蓼	la:n^3^la^6^lja:u^5^	Polygonaceae	Herb	Wild	Whole plant	Decoction; taken orally for hyperosteogeny, abdominal distension, ascites due to cirrhosis, gastric distention, diarrhea, neck lymphatic tuberculosis	451225130718024
*Polygonum Perfoliatum* L.	Gangbangui杠板归	hɣɔk^8^twi^2^khu^5^ta^6^	Polygonaceae	Herb	Wild	Whole plant	Decoction; taken orally for damp and hot jaundice, diarrhea, bacillary dysentery, poor urination, stranguria with turbid discharge, hemorrhoids, eczema, pemphigus, anti-itch	451225000000000
*Polygonum plebeium* R. Br.	Xijianliao习见蓼	pjen^5^jøn^6^	Polygonaceae	Herb	Wild	Whole plant	Decoction; taken orally for pyretic stranguria, jaundice, leukorrhea, depriving ascarid, malnutrition, hemorrhoids, eczema	451225130721005
*Portulaca oleracea* L.	Machixian马齿苋	tɔŋ^6^fan^1^ma^4^	Portulacaceae	Herb	Wild	Whole plant	Decoction; taken orally for bacillary phthisis, diarrhea, bacillary dysentery, fever, cough, internal hemorrhage, eczema	451225130718010
*Pothos chinensis* (Raf.) Merr.	Shiganzi石柑子	–	Araceae	Herb	Wild	Whole plant	Medicinal liquor; taken orally or applied on the affected area, treating for rheumatism, traumatic injury, numbness of meridians and collaterals	451225130308016
*Prunella vulgaris* L.	Xiakucao夏枯草	ha^5^khu^1^hɣɔk^8^	Lamiaceae	Herb	Wild	Infructescence	Decoction; taken orally for scrofula, mammary abscess, breast cancer, dizziness, arthralgia and myalgia, tuberculosis, acute icteric hepatitis, metrorrhagia, leukorrhea. Pounded fresh part applied on the affected area, treating for bleeding wound	451225130420001, 451225130425023
*Psidium guajava* L.	Fanshiliu番石榴	–	Myrtaceae	Tree	both	Leaf, Fruit, Bark	Taken orally directly for stanching bleeding, hepatitis, hepatopathy	451225130724006
Psychotria rubra (Lour.) Poir.	Jiujie九节	mai^4^ta:n^5^lo^4^	Rubiaceae	Shrub	Wild	Stem and leaf	Pounded fresh part applied on the affected area, treating for traumatic injury, bone fracture, rheumatism, ostealgia, swollen toxin, sore throat	451225130307033, 451225130501013, 451225130519040, 451225130608001
*Pteridium aquilinum* (L.) Kuhn var. *latiusculum* (Desv.) Underw. ex Heller	Jue蕨	–	Dennstaedtiaceae	Herb	Wild	Leaf	Medicinal bath for clearing heat and toxic materials	451225130726011
*Pteris vittata* L.	Wugongcao蜈蚣草	–	Pteridaceae	Herb	Wild	Whole plant	Decoction; taken orally for eczema, epilation	451225130727001
*Pteris multifida* Poir.	Jinglanfengweijue井栏凤尾蕨	hɣɔk^8^ci^1^jem^1^	Pteridaceae	Herb	Wild	Whole plant or Root	Decoction; taken orally for abdominal pain, diarrhea, bacillary dysentery, hemafecia, dysuria, urinary urgency. Pounded fresh part applied on the affected area, treating for bleeding wound	451225130311019
*Pterolobium punctatum* Hemsl.	Laohuci老虎刺	–	Fabaceae	Liana	Wild	Root	Decoction; taken orally for hepatitis, duodenal ulcer	451225130727011
*Pueraria montana* (Loureiro) Merrill var. *lobata (*Willdenow) Maesen & S. M. Almeida ex Sanjappa & Predeep	Ge葛	ɔ^6^mε:k^8^ça:u^1^	Fabaceae	Liana	Wild	Rhizome	Decoction; taken orally for alleviate a hangover, vertebral syndrom, clearing away heat and relieving exterior syndrome, stimulate saliva and reduce thirst, measles, diarrhea	451225130804006
*Punica granatum* L.	Shiliu石榴	sik^8^ləu^2^ŋɣa^2^	Lythraceae	Tree	Home garden	Peel	Taken orally directly for diarrhea, bacillary dysentery, protracted dysentery, hemafecia, rectal prolapse, leukorrhea, metrorrhagia and metrostaxis, parasitic accumulation abdominal pain	451225130805001
*Pyrrosia lingua* (Thunb.) Farwell	Shiwei石韦	twi^2^hwi^2^	Polypodiaceae	Herb	Wild	Whole plant	Pounded fresh part applied on the affected area, treating for bleeding wound, gunshot wounds. Decoction; taken orally for clearing heat, calculosis, promoting diuresis and relieving stranguria	451225130806004
*Pyrrosia tonkinensis* (Giesenh.) Ching	Zhongyueshiwei中越石韦	–	Polypodiaceae	Herb	Wild	Whole plant	Decoction; taken orally for nephritis, urinary stone	451225131107008
*Pyrrosia calvata* (Baker) Ching	Guangshiwei光石韦	–	Polypodiaceae	Herb	Wild	Whole plant	Medicinal liquor; taken orally for treating rheumatism	451225121205015
*Quisqualis indica* L.	Shijunzi使君子	–	Combretaceae	Liana	both	Seed	Decoction; taken orally for stomachache	451225131107011
*Raphanus sativus* L.	Luobo萝卜	lak^8^pak^8^la:k^8^	Brassicaceae	Herb	Home garden	Seed	Taken orally directly for treating cough, dyspeptic retention and qi stagnatio, bosom frowsty abdominal distension, diarrhea	451225131107013
*Reynoutria japonica* Houtt.	Huzhang虎杖	cəu^3^lɔŋ^2^ta:ŋ^1^	Polygonaceae	Shrub	Wild	Rhizome	Medicinal liquor; taken orally for treating rheumatism, traumatic injury. Decoction; taken orally for damp and hot jaundice, stranguria, leukorrhea, menostasis, postpartum blood stasis. Pounded fresh part applied on the affected area, treating for traumatic injury, burn and scald, malignant sore and tinea	451225130608004
*Rhodomyrtus tomentosa* (Aiton) Hassk.	Taojinniang桃金娘	–	Myrtaceae	Shrub	Wild	Root, Leaf, Fruit	Taken orally directly or Medicinal liquor; taken orally for astringing to stop diarrhea, dispelling wind and activating collaterals	451225130608030
*Rhus chinensis* Mill.	Yanfumu盐肤木	kwa^6^hu^3^mai^4^	Anacardiaceae	Shrub	Wild	Whole plant	Decoction; taken orally or medicinal bath for cool the blood, cough, sore throat, jaundice, night sweat, diarrhea, kerion, carbuncle toxin, head-wind white scaling	451225131107018
*Rhynchosia volubilis* Lour.	Luhuo鹿藿	–	Fabaceae	Liana	Wild	Whole plant	Decoction; taken orally for aid digestion	451225130723004
*Ricinus communis* L.	Bima蓖麻	la:k^8^ma^6^la:k^8^	Euphorbiaceae	Shrub	both	Seed	Decoction after frying; taken orally for carbuncle, pharyngitis, edema, scrofula, constipation. Pounded fresh part applied on the affected area, treating for scabies	451225121230004
*Rohdea japonica* Roth.	Wannianqing万年青	hwa:n^6^mɛ^1^həu^1^	Asparagaceae	Herb	both	Whole plant	Decoction; taken orally for palpitation, pectoralgia, edema, sore throat. Pounded fresh part applied on the affected area, treating for bleeding wound	451225131107022
*Rosa chinensis* Jacq.	Yuejihua月季花	mwa:n^4^ci^5^hwa^1^	Rosaceae	Liana	Home garden	Bud	Decoction; taken orally for irregular menses, leukorrhea. Pounded fresh part applied on the affected area, treating for traumatic injury	451225131107025
*Rosa laevigata* Miehx.	Jinyingzi金樱子	la:k^8^muŋ^3^ta:ŋ^1^	Rosaceae	Liana	Wild	Root, Fruit	Root: Decoction; taken orally or rinsed for spermatorrhea, enuresis, diarrhea, diarrhea, metrorrhagia and metrostaxis, leukorrhea, uterine prolapse, hemorrhoid complicated by anal fistula, scald. Fruit: medicinal liquor; taken orally for treating spermatorrhea, enuresis, frequent micturition, diarrhea due to spleen deficiency, spontaneous sweating, night sweat, metrorrhagia and metrostaxis, leukorrhea, rectal prolapse	451225130517014, 451225130519046
*Rosa multiflora* Thunb.	Yeqiangwei野蔷薇	tshja:ŋ^6^wəi^6^ta:ŋ^1^	Rosaceae	Liana	Wild	Stem, Leaf	Decoction; taken orally or medicinal bath for abscess of lung, diarrhea, arthritis, internal hemorrhage, irregular menses, furuncle, hemorrhoids. Pounded fresh part applied on the affected area, treating for traumatic injury, acariasis	451225131108002
*Rosa* sp.	Meigui玫瑰	məi^6^ji^1^hwa^1^	Rosaceae	Shrub	Home garden	Flower	Taken orally directly for spitting blood, hemoptysis, irregular menses, leukorrhea, diarrhea, mastalgia, swollen toxin	451225131107030
*Rotala rotundifolia* (Buch.-Ham. ex Roxb.) Koehne	Yuanyejiejiecai圆叶节节菜	–	Lythraceae	Herb	Wild	Whole plant	Decoction; taken orally for cough	451225130425036
*Rubia cordifolia* L.	Qiancao茜草	hɣɔk^8^la:n^3^ta:ŋ^1^	Rubiaceae	Herb	Wild	Whole plant	Decoction; taken orally for treating rheumatism, internal hemorrhage, amenorrhea, jaundice, chronic bronchitis. Pounded fresh part applied on the affected area, treating for rheumatoid arthritis, traumatic injury, painful swelling	451225130311033
*Rubus alceifolius* Poir.	Cuyexuangouzi粗叶悬钩子	–	Rosaceae	Liana	Wild	Root, Leaf	Decoction; taken orally or medicinal bath for clearing heat, stanching bleeding, promoting blood circulation for removing blood stasis	451225130608042, 451225130730004
*Rubus corchorifolius* L. f.	Shanmei山莓	–	Rosaceae	Liana	Wild	Root	Decoction; taken orally for ascites due to cirrhosis, prostatitis, tracheitis	451225130308013, 451225130310029, 451225130425022
*Rubus phoenicolasius* Maxim.	Dongxianxuangouzi多腺悬钩子	–	Rosaceae	Liana	Wild	Root	Medicinal liquor; taken orally for treating rheumatism	451225131108010
*Rubus rosifolius* Smith	Kongxinpao空心泡	–	Rosaceae	Herb	Wild	Root	Decoction; taken orally for ascites due to cirrhosis	451225131108017
*Salix babylonica* L.	Chuiliu垂柳	ja:ŋ^6^liu^3^ŋa^5^	Salicaceae	Tree	Home garden	Stem	Decoction; taken orally for rheumatoid arthritis, gonorrhea, gonorrhea, urinary stoppage, infectious hepatitis B, pemphigus, erysipelas, decayed tooth, swelling and aching of gum	451225131109002
*Salvia chinensis* Benth.	Huashuweicao华鼠尾草	twi^2^nən^1^tshøn^1^	Lamiaceae	Herb	Wild	Whole plant	Decoction; taken orally for menostasis, leukorrhea, swelling and pain, costalgia, damp and hot jaundice	451225140504030
*Sambucus chinensis* Lindl.	Jiegucao接骨草	–	Adoxaceae	Herb	Wild	Whole plant	Decoction; medicinal bath for dispelling wind and remove dampness	451225130426004
*Sanguisorba offieinalis* L.	Diyu地榆	tsi^3^phɣə:t^7^kɣa^2^	Rosaceae	Herb	Wild	Root, Rhizome	Decoction; taken orally for internal hemorrhage, diarrhea, anal fistula. Pounded fresh part applied on the affected area, treating for scald	451225140505011
*Sapium sebiferum* (L.) Roxb.	Wujiu乌桕	u^4^tsin^5^	Euphorbiaceae	Tree	Wild	Bark	Pounded fresh part applied on the affected area, treating for furuncle, eczema, pruritus, bleeding wound. Decoction; taken orally for edema, constipation, abdominal distension, eczema	451225130519026
*Sarcandra glabra* (Thunb.) Nakai	Caoshanhu草珊瑚	cəu^3^kwət^7^tsa^2^	Chloranthaceae	Shrub	Wild	Stem and leaf	Decoction; taken orally for sore throat, wind-heat type common cold, diarrhea bacillary dysentery. Pounded fresh part applied on the affected area, treating for rheumatism, traumatic injury	451225130518041
*Sargentodoxa cuneata* (Oliv.) Rehder et E. H. Wilson	Daxueteng大血藤	ça:u^1^phɣə:t^7^lo^4^	Lardizabalaceae	Liana	Wild	Stem	Medicinal liquor; taken orally for treating rheumatism, ostealgia, backache, traumatic injury, beadache due to deficiency of blood, intestines carbuncle	451225131108025
*Saururus chinenisi* (Lour.) Baill.	Sanbaicao三白草	hɣɔk^8^ta:m^1^pɛ:k^8^	Saururaceae	Herb	Wild	Whole plant	Decoction; medicinal bath for damp and hot, edema, stranguria with turbid discharge, leukorrhea, carbuncle, inchacao	451225130426027
*Schefflera heptaphylla* (L.) Frodin	Ezhangchai鹅掌柴	–	Araliaceae	Tree	Wild	Bark, Leaf	Decoction; taken orally or medicinal bath for rheumatism, rheumatoid, sweating	451225121204025, 451225130309015
*Tacca plantaginea* (Hance) Drenth	Lieguoshu裂果薯	nəm^4^lak^8^pak^8^	Dioscoreaceae	Herb	Wild	Rhizome	Decoction; taken orally for gastrosis	451225130422026, 451225130429009
*Scutellaria barbata* D.Don.	Banzhilian半枝莲	mɣa:ŋ^6^ŋa^5^ljen^6^	Lamiaceae	Herb	Wild	Whole plant	Decoction; taken orally for cancer, ascites due to cirrhosis, internal hemorrhage, sore throat, jaundice. Pounded fresh part applied on the affected area, treating for snake bite, carbuncle toxin, traumatic injury	451225130718028
*Scutellaria baicalensis* Georgi	Huangqin黄芩	–	Lamiaceae	Herb	Wild	Rhizome	Decoction; taken orally for hepatitis, nephritis	451225140504021
*Sedum lineare* Thunb.	Fojiacao佛甲草	hɣɔk^8^fu^6^cap^7^	Crassulaceae	Herb	both	Whole plant	Decoction; taken orally for sore throat, jaundice, diarrhea. Pounded fresh part applied on the affected area, treating for abscess, furuncle, erysipelas, scald, snake bite	451225130422042
*Sedum sarmentosum* Bge.	Chuipencao垂盆草	hɣɔk^8^pa:i^2^pən^2^	Crassulaceae	Herb	Wild	Whole plant	Decoction; taken orally for damp and hot jaundice. Pounded fresh part applied on the affected area, treating for skin and external diseases, snake bite, scald	451225140506013
*Selaginella tamariscina* (Beauv.) Spring.	Juanbai卷柏	kon^3^pɛ:k^8^	Selaginellaceae	Herb	Wild	Whole plant	Decoction; taken orally for stimulating saliva, internal hemorrhage, cough, asthma, jaundice, leukorrhea, stranguria. Pounded fresh part applied on the affected area, treating for edema, scald	451225140508012
*Selaginella uncinata* (Desv.) Spring.	Cuiyuncao翠云草	tshəi^4^jyn^6^hɣɔk^8^	Selaginellaceae	Herb	Wild	Whole plant	Decoction; taken orally for jaundice, diarrhea, edema, rheumatoid arthritis, hemoptysis, sore throat, anal fistula, cold sweat. Pounded fresh part applied on the affected area, treating for bleeding wound, scald	451225121230022, 451225130311029, 451225130612003
*Semiaquilegia adoxoides* (DC.) Mak.	Tiankui天葵	mən^1^khwəi^6^la:k^8^	Ranunculaceae	Herb	Wild	Whole plant	Decoction; taken orally for cough, asthma, edema, stranguria. Pounded fresh part applied on the affected area, treating for furuncle and carbuncle, traumatic injury	451225130306014
*Semiliquidambar cathayensis* H. T. Chang	Banfenghe半枫荷	–	Altingiaceae	Tree	Wild	Root, bark	Medicinal liquor; taken orally or applied on the affected area, treating for rheumatoid arthritis, lumbar muscle degeneration	451225131109028
*Senecio scandens* Buch.-Ham. ex D. Don	Qianliguang千里光	ça:u^1^jɛ^5^	Asteraceae	Herb	Wild	Whole plant	Decoction; taken orally for rhinitis, hepatitis, clearing away heat and toxic materials, sore throat, swelling and pain, pruritus	451225121204007
*Senna occidentalis* (L.) Link	Wangjiangnan望江南	moŋ^6^ca:ŋ^1^na:m^2^	Fabaceae	Herb	Wild	Stem, Leaf	Decoction; taken orally for cough, asthma, bloody stranguria, constipation, headache, red eyes. Pounded fresh part applied on the affected area, treating for furuncle swollen toxin, snake bite	451225140507003
*Senna tora* (L.) Roxb.	Jueming决明	–	Fabaceae	Herb	both	Seed	Decoction; taken orally for clearing liver and eyesight, diuresis, anti-hypertensive	451225131107001
*Serissa japonica* (Thunb.) Thunb.	Liuyuexue六月雪	–	Rubiaceae	Shrub	Wild	Root	Decoction; taken orally for nephropathy, pharyngitis, urinary stone	451225140508032
*Sesamum indicum* L.	Zhima芝麻	yəu^6^ma^2^nam^1^	Pedaliaceae	Herb	Home garden	Seed	Powdered, taken orally with water for premature graying hair, dizzy of the head and dim of sight, constipation due to intestinal dryness	451225130726012
*Setaria italica* (L.) Beauv. var. *germanica* (Mill.) Schrad.	Su粟	–	Poaceae	Tree	Home garden	Stigma, Pulp of infructescence	Decoction; taken orally for diuresis stranguria, removing jaundice detumescence	451225130306003
*Sigesbeckia pubescens* (Makino) Makino	Xiangengxixian腺梗豨莶	çi^1^tshjen^1^hɣɔk^8^	Asteraceae	Herb	Wild	Whole plant	Decoction; taken orally or medicinal bath for rheumatism, numbness of limbs, apoplexy, abscess, eczema pruritus, hypertension	451225130429025
*Smilax biumbellata* T. Koyama	Xinanbaqia西南菝葜	ça:u^1^cəm^1^ka:ŋ^1^	Smilacaceae	Liana	Wild	Rhizome	Medicinal liquor; taken orally or applied on the affected area, for treating dispelling wind and remove dampness, rheumatism, traumatic injury, scrofula	451225130423029
*Smilax china* L.	Baqia菝葜	–	Smilacaceae	Liana	Wild	Tuber	Medicinal liquor; taken orally for tonifying kidney, general fatigue, cough	451225130430025
*Smilax glabra* Roxb.	Tufuling土茯苓	hu^3^kɔk^7^ta:ŋ^1^	Smilacaceae	Liana	Wild	Rhizome	Decoction; taken orally for syphilis, turbidity, inchacao, furuncle, abscess, scrofula	451225130501042, 451225130611002
*Solanum melongena* L.	Qie茄	ca^6^la:i^4^ta:ŋ^1^	Solanaceae	Herb	Home garden	Root, Stem	Decoction; taken orally or medicinal bath for rheumatic arthritis, protracted dysentery, hemafecia, inchacao. Pounded fresh part applied on the affected area, treating for chilblain, toothache	451225131109029
*Solanum nigrum* L.	Longkui龙葵	tɔŋ^6^taŋ^1^lɔŋ^2^	Solanaceae	Herb	Wild	Whole plant	Decoction; taken orally for clearing away heat and toxic materials, diuresis detumescence, anticancer. Pounded fresh part applied on the affected area, treating for furuncle, traumatic injury	451225130307003
*Solanum verbascifolium* L.	Jiayanyeshu假烟叶树	jen^1^ja^4^fa^5^	Solanaceae	Shrub	Wild	Whole plant	Decoction; taken orally or medicinal bath for rheumatism, toothache, scrofula, metrorrhagia. Pounded fresh part applied on the affected area, treating for traumatic injury, furuncle abscess, eczema	451225121205029
*Solena heterophylla* Lour.	Maogua茅瓜	–	Cucurbitaceae	Liana	Home garden	Whole plant	Pounded fresh part applied on the affected area or medicinal bath, treating for pedal edema	451225130606028
*Solidago decurrens* Lour.	Yizhihuanghua一枝黄花	nə^5^ŋa^5^ŋa:n^3^hwa^1^	Asteraceae	Herb	Wild	Whole plant	Decoction; taken orally for common cold with headache, sore throat, cough, jaundice; medicinal bath, treating for infantile convulsions, traumatic injury, furuncle, eczema itch	451225130307026
*Sophora flavescens* Alt.	Kucan苦参	sən^5^kam^1^	Fabaceae	Herb	both	Root	Medicinal liquor; taken orally for treating rheumatism, ostealgia. Decoction; taken orally or medicinal bath for infantile malnutrition, toxic heat, discharging fresh blood stool, jaundice, leukorrhea, acute tonsillitis, hemorrhoid complicated by anal fistula, rectal prolapse, pruritus, scald	451225130719007
*Sophora japonica* L.	Huai槐	hwa:i^6^mai^4^hwa^1^	Fabaceae	Shrub	Home garden	Flower	Decoction; taken orally for piles, internal hemorrhage, hypertension. Stewed with tail of pig intestine and drunk the soup, treating for syphilis	451225130428048
*Sophora tonkinensis* Gagnep.	Yuenanhuai越南槐	–	Fabaceae	Shrub	Wild	Root	Taken orally directly for sore throat, gastric cancer, stomachache, gastric ulcer, prostatitis, diuresis	451225130311063
*Spatholobus suberectus* Dunn.	Mihuadong密花豆	ça:u^1^ci^1^phɣə:t^7^	Fabaceae	Liana	Wild	Stem	Medicinal liquor; taken orally for treating dizziness due to deficiency of blood, soreness of waist, paralysis, irregular menses	451225130429024
*Speranskia cantonensis* (Hance) Pax et Hoffm.	Guangdongdigouye广东地构叶	–	Euphorbiaceae	Shrub	Wild	Whole plant	Decoction; medicinal bath, treating for ague or fever	451225130428050
*Spirodela polyrhiza* (Linnaeus) Schleiden	Ziping紫萍	ȵai^3^	Araceae	Herb	Wild	Whole plant	Decoction; taken orally or medicinal bath for affection of exogenous wind-heat, measles, pruritus, edema	451225130429034
*Stachytarpheta jamaicensis* (L.) Vahl	Jiamabian假马鞭	–	Verbenaceae	Herb	Wild	Whole plant	Decoction; taken orally for clearing away heat and toxic materials, diuresis stranguria, stone, urinary tract infection	451225131108026
*Stahlianthus involucratus* (King ex Bak.) Craib	Tutianqi土田七	–	Zingiberaceae	Herb	Home garden	Tuber	Stewed with meat and eated directly for bodily weakness	451225130607010
*Stellaria alsine* Grimm	Queshecao雀舌草	–	Caryophyllaceae	Herb	Wild	Whole plant	Decoction; taken orally for clearing away heat and toxic materials, diuresis, detumescence relieve pain	451225130307021
*Stemona tuberosa* Lour.	Dabaibu大百部	–	Stemonaceae	Herb	Wild	Rhizome	Decoction; taken orally for relieving thirst and asthma, insecticidal relieve pain, cough with lung heat, tuberculosis	451225130423020
*Stephania japonica* (Thunb.) Miers	Qianjinteng千金藤	ça:u^1^thjen^1^can^1^	Menispermaceae	Liana	Wild	Stem or root, Leaf	Decoction; taken orally for sore blister, diarrhea, rheumatism, edema, stranguria with turbid discharge, sore throat, abscess, furuncle	451225130805003
*Striga asiatica* (L.) O. Ktze.	Dongjiaojin独脚金	na:u^3^tin^1^cəm^1^	Orobanchaceae	Herb	Wild	Whole plant	Decoction; taken orally for infantile malnutrition, edema	451225130721002
*Strobilanthes cusia* (Nees) Kuntze	Banlan板蓝	lo^4^həu^1^fa^5^	Acanthaceae	Herb	Wild	Stem and leaf	Pounded fresh part applied on the affected area, treating for protrusion of lumbar intervertebral disc, snake bite, traumatic injury. Decoction; taken orally or medicinal bath for fever, headache, sore throat, mumps, furuncle, miliaria, eczema	451225130726001
*Strychnos nux*-*vomica* L.	Maqianzi马钱子	–	Loganiaceae	Shrub	both	Seed	Chewing, treating for toothache	451225130501039
*Talinum paniculatum* (Jacq.) gaertn.	Turencan土人参	tɔŋ^6^ma^1^pi^2^	Talinaceae	Herb	Wild	Whole plant	Stewed with meat and drunk the soup, treating for weakness of spleen and stomach, poor appetite, cough hemoptysis, spontaneous sweating, palpitation, irregular menses	451225130422044
*Taraxacum mongolicum* Hand.-Mazz.	Pugongying蒲公英	ma^1^pa:k^8^hɣɔk^8^	Asteraceae	Herb	Wild	Whole plant	Decoction; taken orally or medicinal bath for treating toxic heat, abscess of lung, intestinal carbuncle, mammary abscess	451225130519001
*Taxillus chinensis* (DC.) Danser	Guangjisheng广寄生	–	Loranthaceae	Shrub	Wild	Whole plant	Medicinal liquor; taken orally for rheumatism, activate collaterals, lumbar muscle degeneration, paralysis	451225130424027
*Taxus wallichiana* Zucc. var. *mairei* (Lemée et H. Lév.) L. K. Fu et Nan Li	Nanfanghongdongshan南方红豆杉	–	Taxaceae	Tree	both	Stem pith	Decoction; taken orally for heart disease, hepatopathy	451225131108003
*Tetrastigma planicaule* (Hook. f.) Gagnep.	Biandanteng扁担藤	ça:u^1^pjɛn^3^	Vitaceae	Liana	Wild	Stem, Root	Medicinal liquor; taken orally or rinsed, treating for ischialgia, rheumatism, hemiplegia	451225130727009
*Teucrium viscidum* Bl.	Xuejianchou血见愁	–	Lamiaceae	Herb	Wild	Whole plant	Decoction; taken orally for difficult labour	451225130718001
*Tinospora sagittata* (Oliv.) Gagnep.	Qingniudan青牛胆	–	Menispermaceae	Liana	Wild	Rhizome	Decoction; taken orally for hepatitis, prostatitis. Taken orally directly for abdominal pain	451225130423033
*Tinospora sinensis* (Lour.) Merr.	Zhonghuaqingniudan中华青牛胆	ça:u^1^hɣa:ŋ^4^cen^1^	Menispermaceae	Liana	Wild	Stem	Pounded fresh part applied on the affected area, treating for breaking of muscle and tendon, rheumatism, ostealgia. Medicinal liquor; taken orally for activate collaterals	451225121231021
*Toddalia asiatica* (L.) Lam.	Feilongzhangxue飞龙掌血	lən^1^phɣə:t^7^ta:n^5^	Rutaceae	Liana	Wild	Root or Root bark	Medicinal liquor; taken orally for treating rheumatism, traumatic injury. Powder and applied on the affected area, treating for bleeding wound	451225121205031
*Toona sinensis* (Juss.) Roem.	Xiangchun香椿	mai^4^jam^4^ŋɣa^2^pa:k^8^	Meliaceae	Tree	both	Bark or Root bark	Decoction; taken orally for chronic diarrhea, protracted dysentery, hemorrhoidal hemafecia, metrorrhagia and metrostaxis, leukorrhea, spermatorrhea, gonorrhea, malnutrition, depriving ascarid, tinea sores	451225130611026
*Torilis scabra* (Thunb.) DC.	Qieyi窃衣	–	Apiaceae	Herb	Wild	Seed	Stewed with pork liver and taken orally directly for treating blurred vision, heloma	451225130420004
*Toxicodendron succedaneum* (L.) Kuntze	Yeqi野漆	–	Anacardiaceae	Tree	Wild	Leaf	Decoction; Medicinal bath for treating dermatitis	451225130311047
*Toxicodendron vernicifluum* (Stokes) F. A. Barkley	Qi漆	–	Anacardiaceae	Tree	both	Leaf	Decoction; Medicinal bath for treating dermatitis	451225130519035
*Trachelospermum jasminoides* (Lindl.) Lem.	Luoshi络石	lɔ^6^twi^2^ça:u^1^	Apocynaceae	Liana	Wild	Stem and leaf	Medicinal liquor; taken orally or applied on the affected area, treating for rheumatism, traumatic injury, muscle and vessel contracture etc syndromes, swelling and pain. Decoction; taken orally for spitting blood, postpartum lochia	451225121205021, 451225130429013
*Tradescantia pallida* (Rose) D. R. Hunt	Zizhumei紫竹梅	–	Commelinaceae	Herb	Wild	Whole plant	Medicinal bath for sterilization and anti-itch	451225130607017
*Trichosanthes kirilowii* Maxim.	Gualou栝楼	thjen^1^la^2^pən^5^	Cucurbitaceae	Liana	both	Root	Root: Decoction; taken orally for cough with lung heat, jaundice, pemphigus; Fruit: Decoction; taken orally for cough, palpitation, costalgia, marasmus, frequent micturition	451225130518025
*Tripterygium wilfordii* Hook. f.	Leigongteng雷公藤	ça:u^1^ləi^6^pɣa^3^	Celastraceae	Liana	Wild	Root, Leaf, Flower	Medicinal liquor; Applied on the affected area, treating for scabies, eczema, rheumatic arthritis	451225130309033
*Tupistra* sp.	Kaikoujian开口箭	–	Asparagaceae	Herb	both	Whole plant	Pounded fresh part applied on the affected area, treating for bone injury	451225131108040
*Typha domingensis* Persoon	Changbaoxiangpu长苞香蒲	pu^2^ŋa:n^3^	Typhaceae	Herb	Wild	Whole plant	Decoction; taken orally for internal hemorrhage, metrorrhagia and metrostaxis, dysmenorrhea, menostasis, postpartum blood stasis, bloody stranguria	451225130608027
*Uncaria rhynchophylla* (Miq.) Miq. ex Havil.	Gouteng钩藤	ça:u^1^kau^1^	Rubiaceae	Liana	Wild	Hooked stem	Hooked stem: Decoction; taken orally for blood pressure lowering, epilepsy, dizziness. Root: Medicinal liquor; taken orally for treating rheumatism	451225130430001
*Urena lobata* L.	Ditaohua地桃花	–	Malvaceae	Shrub	Wild	Root, Leaf	Decoction; taken orally for bacillary phthisis, cough, anti-inflammato	451225130427023, 451225130606027
*Verbena officinalis* L.	Mabiancao马鞭草	hɣɔk^8^ma^4^pjen^1^	Verbenaceae	Herb	Wild	Whole plant	Decoction; taken orally for commom cold, tonsillitis, acute nephritis, sore throat, damp and hot jaundice. Pounded fresh part applied on the affected area, treating for mastitis, edema, rheumatoid arthritis, traumatic injury	451225130421046, 451225130427025
*Vernicia fordii* (Hemsl.) Airy Shaw	Youtong油桐	mai^4^tɔŋ^6^lau^5^	Euphorbiaceae	Tree	both	Whole plant	Decoction; taken orally for scrofula, hemorrhoids, scald, crusted tetter, erysipelas, dyspeptic retention abdominal distension, urinary stoppage and constipation, rheumatism, ostealgia	451225130425010
*Viburnum taitoense* Hayata	Taidongjiasan台东荚蒾	–	Adoxaceae	Shrub	Wild	Stem and leaf	Pounded fresh part with salt applied on the affected area, treating for hyperosteogeny, protrusion of lumbar intervertebral disc, relieve pain, traumatic injury	451225130429025
*Vigna radiata* (L.) Wilczek	Lvdong绿豆	lɔk^8^tau^6^	Fabaceae	Liana	Home garden	Seed	Taken orally directly or pounded fresh part applied on the affected area, treating for polydipsia, carbuncle, crotonism	451225130606005
*Vigna umbellata* (Thunb.) Ohwi et H. Ohashi	Chixiaodong赤小豆	tau^6^la:n^3^niŋ^5^	Fabaceae	Liana	Home garden	Seed	Pounded fresh part applied on the affected area, treating for edema, inchacao, jaundice, toxic heat, carbuncle	451225130723004
*Viola japonica* Langsd.	Litoucao犁头草	hɣɔk^8^kɣo^3^khɣəi^1^	Violaceae	Herb	Wild	Whole plant or Root	Pounded fresh part applied on the affected area, treating for furuncle, acute mastitis, intestinal carbuncle, erysipelas, red eyes, snake bite	451225130306012
*Viscum articulatum* Burm. F.	Bianzhihujisheng扁枝槲寄生	–	Santalaceae	Shrub	Wild	Whole plant	Pounded fresh part with the feet of crab applied on the affected area, treating for fractura	451225130612005
*Vitex negundo* L.	Huangjing黄荆	mai^4^jin^1^la:k^8^	Lamiaceae	Shrub	Wild	Fruit	Decoction; taken orally for commom cold, cough, asthma, rheumatoid arthritis, stomachache, hernia, malaria, anal fistula	451225130729004
*Vitex negundo* L. var. *cannabifolia* (Sieb. et Zucc.) Hand.-Mazz.	Mujing牡荆	–	Lamiaceae	Shrub	Wild	Stem	Decoction; taken orally or medicinal bath for commom cold, cough, rheumatism, eliminating stagnation	451225130729006
*Vitis balansana* Planchon	Xiaoguoputao小果葡萄	pɣa^2^pɣa^1^məm^4^	Vitaceae	Liana	Wild	Tendril	Medicinal liquor; taken orally or rinsed, for treating swollen sore, traumatic injury, rheumatism	451225130309044
*Vitis heyneana* Roem. et Schult.	Maoputao毛葡萄	ça:u^1^lak^8^jyt^7^ja^4^	Vitaceae	Liana	Wild	Stem	Medicinal liquor; taken orally or applied on the affected area, treating for rheumatoid arthritis, traumatic injury, sore swollen toxin	451225130722008
*Vitis vinifera* L.	Putao葡萄	–	Vitaceae	Liana	Home garden	Fruit	Taken orally directly for anti-inflammatory, hepatopathy, hepatitis	451225130518017
*Wikstroemia indica* (L.) C. A. Mey	Liaogewang了哥王	–	Thymelaeaceae	Shrub	Wild	Stem and leaf	Decoction; medicinal bath for killing parasites to relieve itshing	451225130428014
*Xanthium sibiricum* Patr. ex Widd	Cangdong苍耳	hɣɔk^8^tsha:ŋ^5^khɣa^1^	Asteraceae	Herb	Wild	Seed, Stem, Leaf	Seed: Decoction; taken orally or medicinal bath for anemofrigid headache, nasosinusitis, rheumatoid arthritis, scabies, pruritus. Stem and leaf: Decoction; medicinal bath, treating for rheumatoid arthritis	451225130607021
*Zanthoxylum nitidum* (Roxb.) DC.	Liangmianzhen两面针	–	Rutaceae	Shrub	Wild	Whole plant	Medicinal liquor; taken orally for rheumatism, relieve pain. Decoction; taken orally for gastric ulcer, stomachache, prostatitis	451225130312018
*Zanthoxylum bungeanum* Maxim.	Huajiao花椒	hwa^5^tsja:u^5^	Rutaceae	Shrub	both	Peel	Stewed with meat and Taken orally directly for invigorating spleen, cold pain in abdomen, vomit, diarrhea, rheumatoid arthritis, colic; taken orally directly for depriving ascarid, enterobiasis; Medicinal bath for pruritus vulvae, pemphigus	451225130421062
*Zea mays* L.	Yushushu玉蜀黍	jəu^6^mɛ^4^mut^8^	Poaceae	Herb	Home garden	Stigmata	Decoction; taken orally for nephrotic syndrome, edema, jaundice, hypertension, damp and hot jaundice, diabetes	451225130518032
*Zephyranthes carinata* Herbert	Jiulian韭莲	–	Amaryllidaceae	Herb	Wild	Bulb	Pounded fresh part applied on the affected area, for stanching bleeding	451225130517016
*Zingiber lingyunense* D. Fang	Wujiang乌姜	–	Zingiberaceae	Herb	Wild	Tuber	Pounded fresh part applied on the affected area, treating for traumatic injury	451225130607040
*Zingiber officinale* Roscoe	Jiang姜	çiŋ^1^khɣɔ^3^	Zingiberaceae	Herb	Home garden	Tuber	Decoction; taken orally or medicinal bath for cough, hemoptysis, hiccough, anemofrigid cold, vomit, cough, reduce phlegm	451225130519031

Mulam name: as written in the international phonetic alphabet (IPA)

Among the families that contributed more medicinal species were Fabaceae and Asteraceae, represented by 29 species (6.36%) in each family, Lamiaceae with 21 species (4.61%), Rosaceae with 16 species (3.51%), Poaceae with 15 species (3.29%), Euphorbiaceae with 14 species (3.07%), Rubiaceae with 13 species (2.85%), and Rutaceae with ten species (2.19%). The other 309 species (67.76%) came from 124 families that were mostly represented by one or two species (Table 3).

**Table 3:** Taxonomic diversity of medicinal plants in the study area

Family	Number of medicinal plant species	Percentage of species (%)	Number of genera	Percentage of genus (%)
Asteraceae	29	6.36	20	5.71
Fabaceae	29	6.36	21	6.00
Lamiaceae	21	4.61	15	4.29
Rosaceae	16	3.51	10	2.86
Poaceae	15	3.29	15	4.29
Euphorbiaceae	14	3.07	9	2.57
Rubiaceae	13	2.85	12	3.43
Rutaceae	10	2.19	6	1.71
Amaranthaceae	9	1.97	6	1.71
Cucurbitaceae	9	1.97	9	2.57
Moraceae	9	1.97	3	0.86
Malvaceae	8	1.75	6	1.71
Polygonaceae	8	1.75	3	0.86
Amaryllidaceae	7	1.54	4	1.14
Vitaceae	7	1.54	4	1.14
Apiaceae	6	1.32	6	1.71
Araceae	6	1.32	6	1.71
Asparagaceae	6	1.32	6	1.71
Polypodiaceae	6	1.32	4	1.14
Solanaceae	6	1.32	3	0.86
Zingiberaceae	6	1.32	4	1.14
Acanthaceae	5	1.10	4	1.14
Berberidaceae	5	1.10	4	1.14
Lauraceae	5	1.10	4	1.14
Menispermaceae	5	1.10	4	1.14
Orchidaceae	5	1.10	4	1.14
Primulaceae	5	1.10	2	0.57
Anacardiaceae	4	0.88	3	0.86
Apocynaceae	4	0.88	3	0.86
Araliaceae	4	0.88	3	0.86
Brassicaceae	4	0.88	4	1.14
Celastraceae	4	0.88	3	0.86
Dioscoreaceae	4	0.88	2	0.57
Myrtaceae	4	0.88	3	0.86
Oleaceae	4	0.88	2	0.57
Phyllanthaceae	4	0.88	3	0.86
Urticaceae	4	0.88	4	1.14
Verbenaceae	4	0.88	4	1.14
Others	142	31.14	122	34.86
Total	456	100.00	350	100.00

### Habit, plant parts used, and habitat

**Fig. 3:**
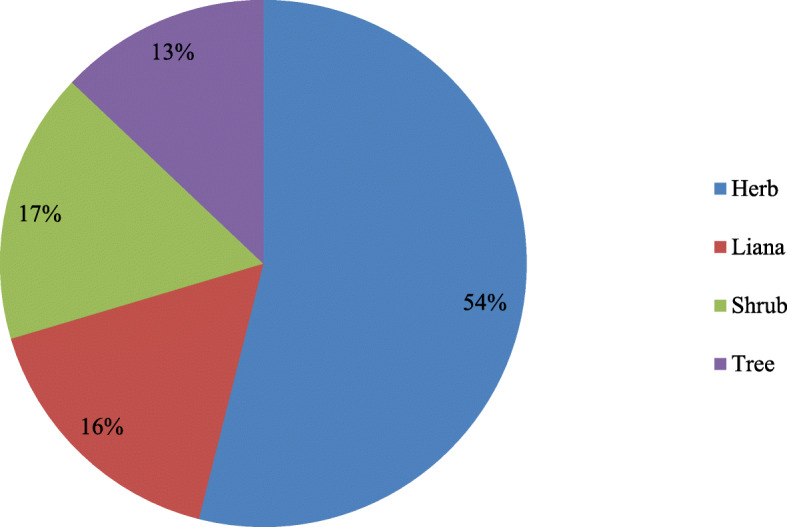
Habits of medicinal plants in the study area

The results of the habit analysis of the medicinal plants showed that herbaceous plants constituted the highest proportion (246 species (54%)), while there were 76 (17%) shrubs, 75 (16%) lianas, and 59 (13%) tree species (Fig. 3).

**Fig. 4:**
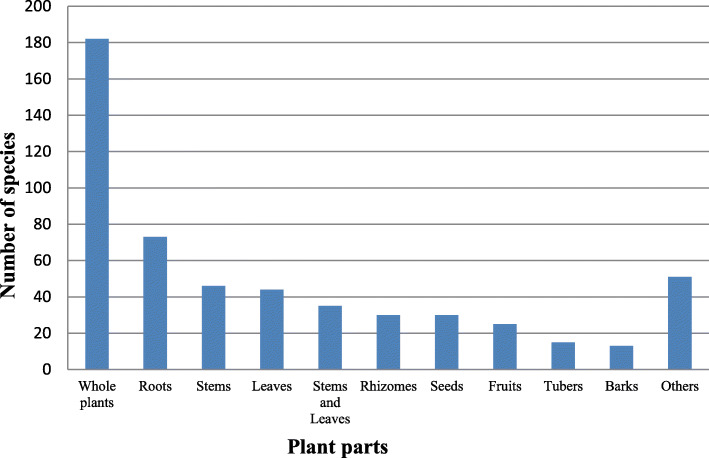
Plant parts used in the treatment of human ailments

Mulam people use different plant parts in the preparation of traditional drugs (e.g., leaves, stems, roots, seeds, bark, flowers, and fruits). Many of the herbal medicines are made by using whole plants (182 species, 33.46%), followed by roots (73 species, 13.42%), stems (46 species, 8.46%), leaves (44 species, 8.09%), a combination of stems and leaves (35 species, 6.43%), rhizomes (30 species, 5.51%), seeds (30 species, 5.51%), fruits (25 species, 4.60%), tubers (15 species, 2.76%), bark (13 species, 2.39%), and 26 other plant parts (e.g., bulbs, flowers, root bark, aril, stigma; 16%) (Fig. 4).

**Fig. 5:**
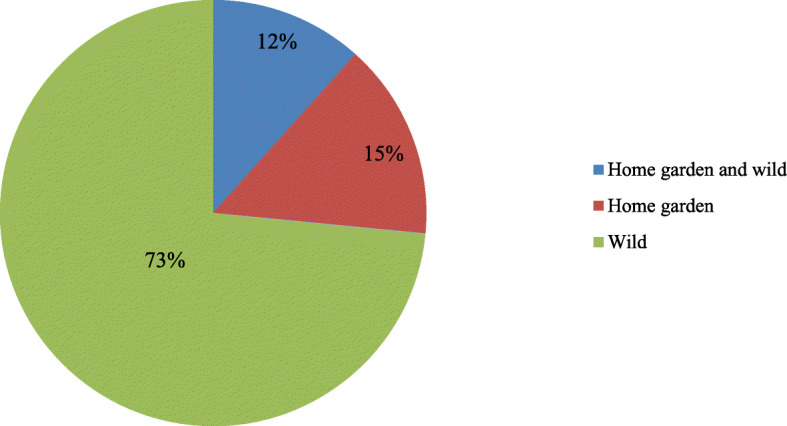
Habitats of medicinal plants in the study area

A total of 456 species of medicinal plants were collected from the study area, most of which (335 species, 73.47%) were obtained from wild habitats; 68 (14.91%) species were from home gardens, and 53 (11.62%) species were both from home gardens and wild habitats (Fig. 5).

### Preparation and application methods

There are numerous different ways to prepare medicinal plants to treat human ailments. In the study area, the most common methods of preparation of traditional medicines from plant material were decoction (54.11%), followed by pounding (20.48%), preparing a medicinal liquor (9.64%), raw (9.64%), stewing (2.75%), and others (Table 4).

**Table 4:** Ways of preparation of medicinal plants

Method of preparation	Frequency	Percentage
Decoction	316	54.11
Pounded	119	20.48
Medicinal liquor	56	9.64
Natural	56	9.64
Stewed	16	2.75
Others (powdered, drying, frying, slicing)	18	3.48

Table 5 shows that the traditional medicines are used in four main ways. The most common method is oral administration (390 plant species, 62.70%), followed by external application (143 species, 22.99%), a medicated bath or rinsing (87 species, 13.99%), and chewing (two species, 0.32%).

**Table 5:** Application method by local Mulam people

Application method	Frequency	Percentage
Oral taking	390	62.70
External application	143	22.99
Medicinal bath or rinsed	87	13.99
Chewing	2	0.32

### Diseases treated in the study area

Based on our investigation and records, medicinal plants were used to treat 312 human ailments in the study area. Based on the statistical analysis, rheumatism was the most common disease treated with 84 medicinal plant species, followed by traumatic injury (71 species), cough (68 species), diarrhea (54 species), jaundice (47 species), abscesses (42 species), furuncles (38 species), edema (36 species), sore throat (34 species), carbuncles (33 species), and eczema (30 species).

### Ranking and informant consensus factor of medicinal plants

Among all of the ailments in the study area, rheumatism was the most common disease and was treated by a high number of medicinal plants (82 species). Ten medicinal plant species were used effectively to treat rheumatism according to key informants. The results revealed that *Semiliquidambar cathayensis* was the most preferred medicinal plant for rheumatism, followed by *Tetrastigma planicaule*, *Bauhinia championii*, and *Millettia lasiopetala* (Table 6).

**Table 6:** Preference ranking to medicinal plants used to treat rheumatism

List of medicinal plants	Informants										Total	Rank
	R_1_	R_2_	R_3_	R_4_	R_5_	R_6_	R_7_	R_8_	R_9_	R_10_		
*Ardisia crenata*	2	1	2	1	6	9	5	4	5	2	37	8
*Ardisia gigantifolia*	5	4	5	2	5	6	4	5	3	4	43	6
*Bauhinia championi*	9	5	6	9	4	8	10	9	9	5	74	3
*Cibotium barometz*	3	3	4	3	3	3	1	3	4	3	30	9
*Clerodendrum japonicum*	4	7	1	8	10	1	3	2	1	1	38	7
*Kadsura coccinea*	6	6	7	4	2	5	7	8	6	8	59	5
*Maclura cochinchinensis*	1	2	3	5	1	2	2	1	2	7	26	10
*Millettia lasiopetala*	7	8	10	6	7	4	6	6	7	9	70	4
*Semiliquidambar cathayensis*	10	9	9	10	9	10	9	7	10	10	93	1
*Tetrastigma planicaule*	8	10	8	7	8	7	8	10	8	6	80	2

R represented respondents; scores in the table indicated ranks given to medicinal plants based on their scarcity. Highest number (10) is for the medicinal plants which informants thought most preferred in the area and the lowest number (1) for the least preferred medicinal plant

Twelve ailment categories were identified based on the eight systems of the human body and the medication characteristics of the Mulam people. The ICF was calculated for each ailment category, and the range was from 0.51 to 0.92 (Table 7). The highest ICF (0.92) was reported for gynecological ailments, with 12 species and 138 use reports, followed by nerves and psychosomatic problems (0.90), digestive system diseases (0.89), urinary system diseases (0.88), skin diseases (0.88), and circulatory system diseases (0.88).

**Table 7:** Informant consensus factor by categories of diseases in the study area

Category	Specific conditions	nur	nt	ICF
Gynecological aliments	Leukorrhea (28), metrorrhagia and metrostaxis (8), irregular menses (12), dysmenorrhea (9), postpartum blood stasis (5), etc.	138	12	0.92
Nerves and psychosomatic problems	Headache (17), insomnia (10), dizziness (8), hemiplegia (5), etc.	83	9	0.90
Digestive system	diarrhea (54), jaundice (47), abdominal pain (18), stomachache (19), abdominal distension (18), constipation (17), etc.	314	36	0.89
Urinary system	diuresis (21), stranguria (19), calculosis (17), urinary frequency (13), dysuria (5), etc.	105	13	0.88
Skin diseases	abscess (42), pruritus (42), furuncle (38), eczema (30), scald (19), inchacao (12), piles (11), scabies (9), etc.	233	28	0.88
circulatory system	internal hemorrhage (25), clearing away heat and toxic materials (23), hypertension (13), hemoptysis (15) , etc.	124	16	0.88
Respiratory system	cough (68), sore throat (34), common cold (30), abscess of lung (8), etc.	189	42	0.78
Traumatic injury and sprain and bleeding wound	traumatic injury (71), bleeding wound (32), bone fracture (7), wound infection, etc.	129	33	0.75
Inflammation	nephritis (16), prostatitis (9), enteritis (10), tracheitis (10), erysipelas (9), cancer (7), dermatitis (5), gastritis (5), pneumonia (5), etc.	129	33	0.75
Rheumatic problems	Rheumatism (60), rheumatoid arthritis (24), etc.	92	43	0.53
Strong body and relieve pain	numbness of limbs (6), backache (8), soreness and weakness of waist and knees (4), stop pains (8), etc.	71	35	0.51
Other Uses	edema (36), male problems (36), pediatric disease (22), scrofula (15), toothache (8), hyperosteogeny (7), etc.	152	36	0.77

### Fidelity levels of most commonly used plants by key informants

For each of the 15 most commonly used plant species as ranked by key informants, the fidelity level (FL) (Table 8) was calculated to quantify their importance in treating a major ailment [31, 35]. The results showed a high FL of greater than 50% for 12 plant species, which highlights the importance of these species in the treatment of the frequently mentioned diseases in the study area. *Polygonum multiflorum*, *Semiliquidambar cathayensis*, *Zingiber officinale*, and *Striga asiatica* had FLs of 100% for strengthening the body and treating rheumatism, infantile malnutrition and cough.

**Table 8:** Fidelity Levels (FL) of most commonly used plants by key informants

Plant species	Therapeutic uses	*I*_p_	*I*_u_	FL%
*Artemisia carvifolia*	Malaria	11	33	33.33
*Camellia oleifera*	Scald	16	25	64.00
*Curculigo orchioides*	Impotence	12	21	57.14
*Eriobotrya japonica*	cough	36	38	94.74
*Gynostemma pentaphyllum*	Anti-inflammatory	34	42	80.95
*Lygodium japonicum*	Renal calculus	16	28	57.14
*Polygonum multiflorum*	Premature graying of the hair	39	39	100.00
*Pueraria montana* var. *lobata*	Hangover alleviation	16	44	36.36
*Ricinus communis*	Scabies	12	18	66.67
*Rosa laeuigata*	Spermatorrhea	18	44	40.91
*Sarcandra glabra*	Common cold	36	43	83.72
*Semiliquidambar cathayensis*	Rheumatism	35	35	100.00
*Sophora tonkinensis*	Stomachache	23	25	92.00
*Striga asiatica*	Malnutritional stagnation	26	26	100.00
*Zingiber officinale*	Cough	42	42	100.00

### Threats to traditional medicinal knowledge and medicinal plants

According to our investigation (Table 1), more than 80% of key informants who showed mastery of rich traditional medicinal knowledge were over 50 years old, and more than 60% of key informants were illiterate or had only received a primary education. Currently, Mulam children spend most of their time in schools, where they receive mainstream culture and education and have no chance to study traditional medicinal knowledge. In addition, young people prefer to look for jobs in urban areas to earn higher incomes. Furthermore, Mulam healers are unwilling to pass on their traditional medicinal knowledge to young people under 30 years old. During our surveys, we found that one-third of doctors did not have a successor. The inheritance process of traditional Mulam medicinal knowledge is experiencing a dilemma. In addition, due to the lack of a written language, basic information on the use of plants, the parts used, drug preparation methods, diseases treated, and other information may be lost or discarded in the transmission process.

According to our field investigation and the group discussions, most of the medicinal plants were found to be under threat from anthropogenic pressure, such as agricultural activities, firewood collection, overgrazing, and logging. Most Mulam villages are located on small strips of flat land or slopes in karst mountainous areas, and most Mulam people engage in traditional agriculture (Fig. 1). Informants ranked agricultural activities as the most serious threat to medicinal plants, followed by firewood collection and overgrazing. The overharvesting of wild medicinal plants was also a key threat because Mulam people prefer to collect whole plants, roots, stems, and rhizomes. This collection method damages or totally destroys the plant and diminishes the sustainability of medicinal plant use.

## Discussion

### Characteristics of informants and their traditional knowledge

Our study included a similar number of men and women as general informants, who have less traditional medicinal knowledge than key informants. Most informants only knew a small number of medicinal plants for treating some common ailments, such as traumatic injuries, abdominal pain, and diarrhea. Every key informant knew more than 60 species and more therapeutic methods for different diseases than the general informants. Most of the key informants were male because Mulam women mainly perform housework and farm work. According to the customary inheritance practice, local traditional medicinal knowledge is typically passed on from an older herbalist to a male successor, rather than a female successor. The number and use methods of medicinal plants reported increased with informant age. Older informants possess more traditional knowledge of medicinal plants than younger people. Local herbalists are unwilling to pass on traditional medicinal knowledge to people who are under 30 years old because they believe that young people are too immature to seriously learn the traditional knowledge. Differences in knowledge of medicinal plants among age and gender groups were also reported in other studies from China and other countries [10, 14, 37, 38].

Most informants in our study have attained low levels of education. Only 33 informants received secondary education, and four informants received tertiary education. Currently, highly educated people tend to prefer modern medicinal technology to traditional knowledge. They are not interested in studying or practicing ethnomedicinal knowledge, especially younger generations. Similar results from other studies also reported that most traditional medicinal herbalists and inheritors worldwide have low formal education levels [10, 15, 17, 22].

### Methods of medicinal plant collection and patient diagnosis and treatment

According to our investigation, local herbalists believe that it is much better to collect medicinal plants from noon to evening in autumn or winter because many medicinal plants may enter dormancy and have relatively dry bodies with the highest efficacy. The herbalists also said that if they met a pregnant woman or someone combing their hair on their way to pick medicinal plants, the collected medicinal plants would have a negative impact on the medication made from the plant. Therefore, the herbalist would not go to collect medicinal plants on that day. They reported that if the first herb were obtained very easily, all of the medicinal plants collected on the same day would have good efficacy. In addition, when Mulam healers collect medicinal plants, there is a tradition of “keeping a line,” that is, they will put money and rice under the roots of the collected plant and leave a few organs rather than collecting the whole plant.

The Mulam herbalists would let their patients rest for 10–20 min to allow their heart rhythm to normalize before feeling their pulse and inquiring about their condition. Many herbalists would diagnose the disease in combination with the hospital’s inspection report. They would ask patients to go to the hospital for a recheck to ensure that the disease would be cured by the end of their therapy. The key informants believed that when patients filled their prescriptions, if the herbalist were smoking or going out with a hoe, the medicine would not be effective. However, if the herbalist were eating or drinking, the medicine would have good efficacy. To prevent their prescriptions from being stolen and to maintain a sense of mystery, the doctors often made the medicines into granules or pills for patients.

### Diversity of medicinal plants

A total of 456 medicinal plant species belonging to 350 genera and 132 families were documented and identified for treating human ailments. Both Fabaceae and Asteraceae (with 29 species) occupied the highest proportion (6.36%), followed by Lamiaceae, Rosaceae, Poaceae, Euphorbiaceae, Rubiaceae, and Rutaceae. Various studies in China showed a similar result, in which these families contain many medicinal species [19, 20, 22, 37, 39]. Most of the families were represented in the study area by one or two species, and the distribution of medicinal plant species in the various families was relatively scattered; this finding reflects the rich biodiversity of the medicinal plants used by Mulam people.

Mulam people believe that wild medicinal plants have stronger efficacy than those from home gardens; therefore, most of the mentioned medicinal plants were harvested from the wild (335 species, 73.47%). Similar findings were reported by other studies from southern China [22, 25, 37, 39]. The herbalists grew a few plants in their home gardens that have multiple uses, are critically endangered in the field, or are urgently needed, such as *Paris polyphylla* var. *chinensis* and *Cynanchum atratum*.

The medicinal plants most widely used by Mulam people were obtained from herbs, which constituted the largest habit category with 246 species (54%). This finding is consistent with other results [37, 39–41]. To explain this phenomenon, Moa et al. suggested that herbs are more widely distributed (roadsides, home gardens, farmlands, and wild habitats) than plants with other habits, such as trees, shrubs, and lianas [30]. In addition, herbs are more easily gathered than tree species [41].

Mulam people like to use whole plants (182 species, 33.46%) in the preparation of traditional drugs, and similar results were found in the neighboring Maonan, Yao, and Zhuang communities [24, 38, 40–42]. The use of roots (73 species, 13.42%), stems (46 species, 8.46%), and rhizomes (30 species, 5.51%) was also common in the study area. However, a clear relationship exists between plant parts collected or the collection method and the impact on the harvested plant [42]. The collection of whole plants, roots, stems, and rhizomes damages or totally destroys the plant and negatively affects the sustainable use of the species. Mulam healers believe that different parts of the same plant may have different medicinal efficacy. The root and stem of *Kadsura longipedunculata*, for example, are decocted and taken orally for gastritis, and a medicinal liquor made from the fruit is taken orally to treat rheumatism and stomachache. The herbalists also reported that different parts of different plants may have the same medicinal purpose. For instance, the stem of *Sargentodoxa cuneata*, the root of *Semiliquidambar cathayensis*, the stem of *Tetrastigma planicaule*, and the whole plant of *Zanthoxylum nitidum* could be used to treat rheumatism.

Mulam healers are skilled at using the principle of “lingqi” and have a tradition of “treating diseases using medicine with a similar shape or color.” The herbalists reported using medicines from hollow-stem plants such as *Equisetum hyemale*, *Siegesbeckia orientalis*, *Leonurus japonicus*, and *Coix lacryma-jobi* var. *ma-yuen* to treat edema based on the aeration of the hollow stems. The branch joints of *Achyranthes bidentata*, *Polygonum capsicum*, and *Taxillus chinensis* are similar to human joints and are often used to treat arthritis. Black soya bean, black sesame seed, mulberry, black ants, and black fungus have black “lingqi” and can be used for treating prematurely white hair.

### Methods of medicinal plant preparation and application

In the study area, various methods used by the local Mulam people for the preparation and administration of medicinal plants were investigated and documented. Decoction (316 species involved, 54.11%) is the most common application method for Mulam people. Mulam people and herbalists believe that decoction accelerates the absorption of medicinal ingredients and improves the taste of medicinal plants. Decoction is cited as the most common method of preparation of herbal remedies and is used widely by other ethnic groups [10, 22, 43–47]. Pounding also had a high frequency (119) and percentage (20.48%).

Mulam people and herbalists prefer to prepare fresh materials directly through decoction or pounding. They believe that the raw medicinal plants possess better efficacy than cooked plants. In addition, the rich plant diversity around Mulam villages provides a material basis for the use of raw medicinal plants. Additionally, the raw material may maintain its volatile oils and other ingredients [22]. However, the utilization of fresh plant parts may threaten the plants due to frequent collection, including in dry seasons [30]. Certain measures and methods should be taken immediately to guide and encourage local people to grow medicinal herbs and to store commonly consumed medicinal materials.

Oral administration (390 species involved, 62.7%) is the most common method of administration of traditional medicine by Mulam people. Oral use was considered popular because it is a simple administration method. It has also been found to be widely applied in other studies [10, 22, 43–47]. Different additives, such as alcohol, honey, salt, and sugar, are widely used by Mulam healers to improve the flavor, taste, and general acceptability of certain orally administered remedies. In addition, Mulam people often stew animal bones, innards, or meat with medicinal plants. Mulam healers believe that animal organs can nourish the corresponding parts of the human body. For example, chicken liver and *Buddleja officinalis*, *Senecio scandens*, and *Centipeda minima* cooked together can be used to treat hepatitis. Pork kidney and *Eucommia ulmoides* and *Allium tuberosum* cooked together are used to improve renal function. They also believe that improving patient nutrition can improve the efficacy of medicinal plants for patients.

Medicinal baths were frequently mentioned during our investigations. Mulam people reported that medicinal baths are safe, simple to perform, and did not result in side effects as an external treatment method. A medicinal bath is usually used for sweating, fever reduction, activating blood circulation to dissipate blood stasis, expelling wind to relieve excess gas, and providing itching relief [18]. Medicinal baths can treat diseases and can also prevent diseases. When taking a medicinal bath, the skin is fully exposed to the medicinal bath water so that the bath constituents with medicinal value can be absorbed [48, 49]. Hot water can also stimulate blood capillaries and metabolism. Medicinal baths are commonly used by the Yao and Zhuang people who live in humid mountainous areas of southern and southwestern China [18, 37, 49–51].

### Diseases, ranking, and informant consensus factor of medicinal plants

Based on our investigations, 312 human ailments are treated with medicinal plants by Mulam people. According to our statistical analysis, rheumatism had the highest number (84 species) of medicinal plants used for its treatment. Mulam people living in humid and mountainous areas engage in heavy manual labor to survive. Thus, rheumatism is the most common disease in the study area. Because of the complexity of rheumatism, its pathogenesis has not been fully clarified [52]. Rheumatism is common all over the world and has been studied by different research institutions and organizations [52–55]. Numerous medicinal plants are used by Mulam herbalists to treat rheumatism. Ten medicinal plant species are widely used to treat rheumatism according to the key informants. In the preference ranking exercise, *Semiliquidambar cathayensis* was the most preferred medicinal plant. *S*. *cathayensis* is mainly used to treat rheumatism, lumbar muscle injury, hemiplegia, traumatic injury, and other conditions [56]. It is a very popular and effective traditional local medicine for rheumatism in Yao communities [37]. Mulam healers prefer to use the roots and bark of *S*. *cathayensis* collected from the wild to treat rheumatism. The large-scale collection of roots and bark threatens the sustainable development of *S*. *cathayensis*. Alternative plant parts or species for treating rheumatism urgently need to be discovered and studied.

Most of the ailment categories had a high ICF value (greater than 0.7), such as gynecological ailments (0.92), nerves and psychosomatic problems (0.90), digestive system ailments (0.89), and urinary system ailments (0.88). The higher the ICF value is, the higher the diversity of plant species used by herbalists to treat the disease. The lower the ICF value is, the lower the number of plant species used by herbalists to treat the disease [31]. The high ICF for gynecological ailments can probably be attributed to the local people preferring to obtain medicinal plants from wild habitats nearby, inheriting traditional medicinal knowledge from their parents or grandparents, and having little communication with other people to prevent others from stealing relevant prescriptions. The category of plants used to strengthen the body and release pain had the lowest degree of consensus (0.51) because most of these medicinal plants are easily obtained and used for multiple purposes, such as foods, vegetables, and tea substitutes.

### Fidelity levels of the most commonly used plants by key informants

*Polygonum multiflorum*, *Semiliquidambar cathayensis*, *Zingiber officinale*, and *Striga asiatica* have the highest fidelity level (FL) values (100.00%). *Eriobotrya japonica* (94.74%) and *Sophora tonkinensis* (92.00%) also have high FL values. The remedies for frequently reported ailments have the highest FL values, and those with a low number of reports have the lowest FL values [36]. Obviously, these medicinal plants were very effective in the treatment of premature hair graying, rheumatism, infantile malnutrition, cough, and stomachache, which are frequently reported in the Mulam district and widely used by Mulam healers. Additionally, *E*. *japonica* (38), *Gynostemma pentaphyllum* (42), *P*. *multiflorum* (39), *Pueraria montana* var. *lobata* (44), *Rosa laevigata* (44), *Sarcandra glabra* (43), and *Z. officinale* (42) have high *I*_u_ values, showing that these medicinal plants were widely applied by Mulam healers and have high medicinal value.

### Comparison with traditional Chinese medicine and previous ethnobotanical studies

To assess the novelty of the ethnomedicinal use of the encountered species, we chose 33 frequently or uniquely used medicinal plant species and compared their use with traditional Chinese medicine (TCM) and previously published reports from neighboring areas of southern China (Table 9) [18–20, 22, 25, 37, 39, 50, 51, 57–66].

**Table 9:** Comparison with traditional Chinese medicine (TCM) and previous ethnobotanical studies

Plant species	Diseases treated by Mulam	Diseases treated in traditional Chinese medicine and previous ethnobotanical studies
*Achyranthes longifolia*	Calculosis	Traumatic injury, rheumatism, dysentery, diphtheria, sore throat, sore carbuncle, stranguria, edema, removing blood stasis, kidney empty lumbago, dysmenorrhea, hypertension
*Acorus gramineus*	Epilepsy, phlegm heat, abdominal distension, abdominal pain, traumatic injury	Epilepsy, phlegm heat, rheumatism, beautifying, bellyache, tummy bug, numbness of limbs, hemorrhoids, diarrhea, gall, injuries from falls, dysmenorrhea
*Artemisia argyi*	Tocolysis, dysmenorrhea, irregular menses, leukorrhea, metrorrhagia and metrostaxis	Irregular menstruation, spitting blood, uterine bleeding, postpartum hemorrhage, carbuncle and scabies, stopping bleeding by warming meridians, expel cold and alleviate pain
*Artemisia carvifolia*	Malaria, diarrhea, jaundice, scab capillaris ies, pruritus	Malaria, sunstroke, dysentery, jaundice, scabies, pruritus
*Camellia oleifera*	Abdominal pain, depriving ascarid, intestinal dryness, scabies, scald	Acute laryngopharyngitis, cold, diarrhea, stomachache, pruritus
*Clerodendrum bungei*	Tuberculosis, carbuncle, furuncle, eczema, piles, rectal prolapse, infantile convulsion	Carbuncle, furuncle, eczema, enriching the blood
*Corydalis saxicola*	Anti-inflammatory	Acute or chronic hepatitis, scabies swelling poison
*Cupressus funebris*	Liver ascites	Children with high fever, vomiting blood, burns, hemorrhoids, dysentery
*Curculigo orchioides*	Impotence, aconuresis, carbuncle, scrofula	Impotence, urinary incontinence, uterine bleeding, ulcer, scrofula, headache due to common cold, rheumatic arthritis, rheumatism, nourishing, strengthening muscles and bones
*Dioscorea bulbifcra*	Antral gastritis, enteritis, thyroid disease, cough with lung heat, pudendal ulcer	Goiter, lymphatic tuberculosis, sore throat, hematemesis, hemoptysis, whooping cough, cancer, sore furuncle, epistaxis, pneumonia
*Eriobotrya japonica*	Ascites due to cirrhosis, cough with lung heat, hemoptysis, clearing away heat and toxic materials	Pertussis, cough, hematemesis, emesis
*Euphorbia esula*	degerming, chills, fever	–
*Ficus hirta*	Stomachache, cough, abdominal distension, edema, leukorrhea, rheumatoid arthritis, lumbago	Consumption, cough, abdominal distention, edema, rheumatism arthralgia, hepatitis, leucorrhea, no milk after delivery
*Flemingia macrophylla*	Caligo of old people	Rheumatic, lumbar muscle strain, hemiplegia and impotence
*Gynostemma pentaphyllum*	Relieve fever, anti-inflammator, chronic tracheitis, cough, asthma, stomachache, insomnia, headache	Cough, chronic gastroenteritis, rheumatism, bronchitis, stomachache
*Hedyotis diffusa*	Lung heat, sore throat, jaundice, pelvic inflammation, carbuncle, snake bite	Appendicitis, sphagitis, jaundice, adverse urination, dysentery, tumors, boils swelling, snake bite, hepatitis, cough, bronchitis, tonsillitis, toothache, cancer
*Laportea violacea*	Ascites due to cirrhosis	Rheumatic arthritis, urticaria, eczema, stomachache, malnutrition, epilepsy, sciatica
*Lygodium japonicum*	Kidney stone, clearing heat and diuresis, stranguria. Pounded fresh part applied on the affected area for anaesthesia	Stranguria, gonorrhea, leukorrhea, hepatitis, sorethroat, enteritis, dysentery, eczema, shingles, hematemesis, bleeding wound, jaundice, itch, diuresis, calculus, rheumatism, chronic ulcer, skin infection, furuncle, foot rot
*Pinus massoniana*	Rheumatic arthritis, tuberculous arthritis, blood stasis, rheumatoid arthritis, traumatic injury, insomnia, edema, eczema, hemorrhoids	Vertigo, stomachache, dysentery, traumatic hemorrhage, eczema, skin erosion, measles
*Polygonum multiflorum*	Insomnia, profuse sweating, skin eruption, kidney deficiency, premature graying of the hair, dizzy of the head and dim of sight, soreness and weakness of waist and knees, spermatorrhea, chronic hepatitis, abscess, constipation due to intestinal dryness	Vertigo, tinnitus, premature graying of the hair, lumbar and knee pain, limb numbness, neurasthenia, hyperlipidemia, carbuncle, rubella, constipation, spermatorrhea, malaria, dysentery, chronic hepatitis, scrofula, intestinal wind, hemorrhoid, kidney deficit, dizziness, insomnia, postpartum bellyache, retention of blood in uteru
*Pueraria montana* var. *lobata*	Alleviate a hangover, vertebral syndrom, clearing away heat and relieving exterior syndrome, stimulate saliva and reduce thirst, measles, diarrhea	Fever, headache, diarrhea, hypertension, stenocardia, epicophosis
*Ricinus communis*	Carbuncle, pharyngitis, edema, scrofula, constipation, scabies	Rheumatoid arthralgia, tetanus, epilepsy, schizophrenia, ulcer, pharyngitis, scrofula, scald, scabies
*Rosa laevigata*	Spermatorrhea, enuresis, diarrhea, diarrhea, metrorrhagia and metrostaxis, leukorrhea, hemorrhoid complicated by anal fistula, scald, frequent micturition, diarrhea due to spleen deficiency, spontaneous sweating, night sweat, leukorrhea, rectal prolapse	Spermatorrhea, enuresis, diarrhea, metrorrhagia and metrostaxis, leukorrhea, uterine prolapse, rectal prolapse, hemorrhoid, scald, spontaneous sweating, night sweat, bone fracture, traumatic injury, appendicitis, enteritis, stomachache
*Sarcandra glabra*	Sore throat, wind-heat type common cold, diarrhea bacillary dysentery, rheumatism, traumatic injury	Rheumatic arthralgia, traumatic injury, fracture, pneumonia, appendicitis, tumour, bacillary dysentery, cholecystitis, abscess, sore throat, rheumatism, promoting blood circulation, heat clearing and detoxifying
*Schefflera heptaphylla*	Rheumatism, rheumatoid, sweating	Fever, rheumatism, traumatic injury, sore throat, for eczema, allergic dermatitis, dermatitis, eczema
*Semiliquidambar cathayensis*	Rheumatoid arthritis, lumbar muscle degeneration	Rheumatism, rheumatoid, traumatic injury, relaxing tendons and activating collaterals, promoting blood circulation, postpartum recovery, skin disease
*Sophora tonkinensis*	Sore throat, gastric cancer, stomachache, gastric ulcer, prostatitis, diuresis	Sorethroat, swelling and aching of gum, jaundice, diarrhea, hemorrhoids, scabies, snake bite, acute pharyngitis, tonsillitis, cough, constipation, clearing heat and detoxifying, diminishing inflammation, relieving pain
*Striga asiatica*	Nfantile malnutrition, edema	Pacify liver and clear heat, remove food retention, infantile malnutrition, dampness-heat constitution, diarrhea, jaundiced hepatitis
*Toddalia asiatica*	Rheumatism, traumatic injury, bleeding wound	Rheumatism, traumatic injury, stomachache, bleeding wound, amenorrhea, algomenorrhea, furuncle, intercostal neuralgia, skin disease, relieving pain, hemiplegia
*Toxicodendron vernicifluum*	Dermatitis	Traumatic injury, traumatic bleeding, sore carbuncle
*Viburnum taitoense*	Hyperosteogeny, protrusion of lumbar intervertebral disc, relieve pain, traumatic injury	–
*Zanthoxylum nitidum*	Rheumatism, relieve pain, gastric ulcer, stomachache, prostatitis	Traumatic injury, rheumatism, stomachache, toothache, snakebite, diarrhea, malaria, chronic gastricism
*Zingiber officinale*	Cough, hemoptysis, hiccough, anemofrigid cold, vomit, cough, reduce phlegm	Cold, vomiting, cough, release superficies, warm the middle, resolve phlegm and stop cough

The comparison showed that the diseases treated with the most frequently used plants by Mulam people were similar to those found in previous ethnobotanical studies and TCM. For example, *Acorus tatarinowii* was the most frequently used plant for epilepsy, phlegm heat, abdominal distension, abdominal pain, and traumatic injury in the study area. Similarly, it is used to treat epilepsy and phlegm heat in TCM [57]. In addition, this plant is used for rheumatism and beautification in the Yao communities of Longsheng County, Northern Guangxi [25], and it is used to treat stomachache, stomach flu, limb numbness, hemorrhoids, diarrhea, gall, injuries from falls, and dysmenorrhea and as an invigorant by Yao people in Jinping County, southeastern Yunnan [50]. In Guangdong, this plant is used to treat flu, detumescence, and pain by Hakka people [59]. There are some similarities and differences in the diseases treated with *A*. *tatarinowii*, and it is used in different places and by different groups of people. However, some unique medicinal plant species (e.g., *Achyranthes longifolia*, *Cupressus funebris*, *Euphorbia esula*, *Flemingia macrophylla*, *Laportea violacea*, *Pinus massoniana*, *Toxicodendron vernicifluum*, *Viburnum taitoense*) had completely novel medicinal functions reported in our study area that had never been reported in other investigations or recorded in TCM. For example, *A*. *longifolia* was reported in the present study as only being used for calculosis, whereas it is used for traumatic injury, rheumatism, dysentery, diphtheria, sore throat, sore carbuncle, stranguria, and edema in TCM [57]. In southern and southwestern China, this plant was used to treat blood stasis, empty-kidney lumbago, sore throat, dysmenorrhea, hypertension, and traumatic injury by Yao and Miao people [20, 58]. *Euphorbia esula* is another species mentioned for the first time. It was reportedly used as a disinfectant and to treat chills and fever in a medicinal bath or by placing it on the patient’s bed. Previous studies conducted in other areas mentioned the use of *Euphorbia* spp. to treat rheumatism, promote blood circulation, cure furuncles, and treat inflammations of unknown origin [22, 61]. *V*. *taitoense* is a *Viburnum* medicinal species mentioned for the first time. It was reported as being used to treat hyperosteogeny, protrusion of the lumbar intervertebral disc, pain, and traumatic injury in the current study. Previous studies conducted in other areas mentioned treatment with *Viburnum* spp. for toxicoderma, rheumatism, traumatic injury, and to stop bleeding [25, 61]. The pharmacological activity of these plants is a novel finding that has only been reported for such medicinal purposes in this area. Our investigation found that traumatic injury, bacterial infection, calculosis, hyperosteogeny, cough, and fever were the most common diseases in Mulam villages. Mulam people are skilled in using plants from their surroundings to treat diseases in their daily lives. They not only make full use of the surrounding plant resources but also continuously communicate and learn from other ethnic groups in their long-term struggle with the natural environment and diseases.

### Threats to traditional medicinal knowledge and medicinal plants

Our investigation and group discussions revealed that traditional medicinal knowledge is greatly threatened due to the lack of a written record, conservative inheritance patterns, and low interest in traditional medicinal knowledge from young people. In addition, agricultural activities, firewood collection, overgrazing, logging, and overharvesting of medicinal plants resulted in a decrease in medicinal plant resources and associated traditional knowledge. Additionally, the superstition and the mystery surrounding the Mulam healers’ traditional medicinal knowledge are also regarded as obstacles to dissemination and promotion. Thus, policies to improve the conservation, development, and sustainable use of Mulam medicinal plants and associated traditional knowledge are essential. First, further investigation and documentation of traditional Mulam medicinal knowledge is imperative. Books and databases of medicinal plants, animals, and minerals should be published, with free access provided to local healers and those (especially young people) who are interested in Mulam ethnomedicine. Second, advanced theories and methods of pharmacology, chemistry, and molecular biology should be applied to study the traditional Mulam medicinal knowledge and enhance Mulam people’s understanding and confidence. Third, it is also necessary to encourage the Mulam people to conserve medicinal plants in situ and ex situ, such as by planting endangered and preferred medicinal species in their home gardens or farmlands.

## Conclusions

A total of 456 medicinal plant species used by Mulam people to treat 312 human ailments were investigated and recorded. This result reflects the rich diversity of medicinal plants in the Mulam area. These medicinal plants play an important role in the Mulam healthcare system. Most of the plants (335 species, 73.47%) were obtained from wild habitats, and the herbaceous habit was the most common growth habit (246 species, 54%). The most common method of administration was oral administration, which was used for 390 species (62.70%), and the most common method of preparation was decoction (316 species, 54.11%). Mulam people are skilled in using the plants in their surroundings to treat diseases in their daily lives. Additionally, they continuously communicate and learn from other ethnic groups in their long-term struggle to survive the natural environment and diseases. However, traditional medicinal knowledge and medicinal plants are greatly threatened by rapid economic development for various reasons. Thus, policies and practices for the conservation of medicinal plants and their associated traditional knowledge are necessary.

## Data Availability

We have already included all data in this manuscript.

## Abbreviations

ICF: Informant consensus factor
FL: Fidelity levels
TCM: Traditional Chinese medicine
